# The burden of dietary risk factors in the Nordic and Baltic countries: a systematic analysis for the Global Burden of Disease Study 2023

**DOI:** 10.1016/j.lanepe.2025.101543

**Published:** 2025-11-25

**Authors:** GBD 2023 Nordic and Baltic Diet Collaborators, Ann Kristin Skrindo Knudsen, Carl Michael Baravelli, Christian Madsen, Benjamin Clarsen, Ayodeji Emmanuel TopeAmobonye, Michael Brauer, Anette Kocbach Bølling, Omid Dadras, Demewoz Haile, Rasmus J. Havmoeller, Anne Høyer-Lund, Nityanand Jain, Lars Johansson, Mikk Jürisson, Joonas H. Kauppila, Adnan Kisa, Mika Kivimäki, Ilari Kuitunen, Javaid Nauman, Gavin Pereira, Tagli Pitsi, Pratik Pokharel, Tommi Juhani Vasankari, Stein Emil Vollset, Eva Warensjö Lemming, Marcin W. Wojewodzic, Rune Blomhoff

**Keywords:** Diet, Deaths, Disability-adjusted life-years (DALYs), Attributable burden

## Abstract

**Background:**

Detailed knowledge about the disease burden from unhealthy diet in Nordic and Baltic countries is lacking. This study quantifies and compares deaths and disability-adjusted life-years (DALYs) from dietary risks in these countries.

**Methods:**

Data from the Global Burden of Disease study 2023 (GBD 2023) was used. Attributable disease burden from 15 dietary risks was analysed using the comparative risk assessment framework. Steps included: (1) estimating dietary intake; (2) assessing relative risks of dietary factors on disease endpoints; (3) determining theoretical minimum risk exposure levels (TMREL); and (4) estimating dietary risk-attributable disease burden as numbers and age-standardised rates (ASR) of deaths and DALYs.

**Findings:**

Across the Nordic and Baltic countries (total population = 34,064,020), dietary risks resulted in 38,450 attributed deaths (95% uncertainty interval 10,749–59,386) and 735,284 DALYs (242,417–1,06,638) in 2023. Leading dietary risks included high intake of processed meat and low intake of fruits and whole grains. Dietary risks accounted for 24.9% of cardiovascular disease burden (5.0–37.6), 29.6% of diabetes and kidney disease burden (18.6–40.0), and 7.8% of neoplasm burden (2.9–12.1), with higher burden in the Baltic countries and Greenland than in the Nordic countries.

**Interpretation:**

A substantial disease burden can be attributed to dietary risks in the Nordic and Baltic countries. Knowledge about the impact from unhealthy diet can inform targeted public health policies.

**Funding:**

Gates Foundation and Norwegian Institute of Public Health.

**Research in context**

**Evidence before this study**

The Global Burden of Diseases, Injuries and Risks Study (GBD) has estimated disease burden attributable to dietary risks since 2015. The GBD 2023 update provided global, regional and national estimates for 15 dietary risks from 1990 to 2023 showing that dietary risks rank between the 2nd and 5th most important risk factor groups for disease burden among the Nordic and Baltic countries. While GBD offers valuable international comparisons, more detailed regional analyses are needed to guide local dietary policy. The Nordic Nutrition Recommendations (NNR), form the scientific basis for national dietary guidelines in the Nordic and Baltic countries. NNR 2023 is the region's most recent and comprehensive review of nutrients intake, diets, and health associations. NNR2023 does not, however, model diet-related mortality and disease burden in these countries. To identify prior studies estimating attributable disease burden from diet-related risk factors in Nordic/Baltic countries or regional level, we searched PubMed from inception to October 2025 using: (“diet” [Title] OR “dietary risk” [Title] OR “nutrition” [Title] OR “food intake” [Title]) AND (“mortality” [Title] OR “disease burden” [Title] OR “DALY” [Title] OR “GBD” [Title]) AND (“Nordic countries” OR “Denmark” OR “Finland” OR “Iceland” OR “Norway” OR “Sweden” OR “Baltic countries” OR “Estonia” OR “Latvia” OR “Lithuania” OR “Greenland”). Only one relevant study was identified, which used macro-simulation to estimate preventable cardiovascular and diet-related cancer deaths from adherence to the NNR. The largest potential gains were associated to higher fruit and vegetable intake, except in Denmark, where lower salt intake yielded the largest benefit. However, this study did not quantify non-fatal outcomes or overall diet-attributable burden. Overall, the existing evidence base indicates substantial data gaps regarding the population-level impact of diet on mortality and disease burden in the Nordic and Baltic countries.

**Added value of this study**

This study provides a comprehensive and comparative overview of intake of 15 dietary risks in Nordic and Baltic countries and quantifies the potential impact of suboptimal intake of each diet component on disease mortality and morbidity among the countries. The Burden of Proof Risk Function methods (BPRF), provides supplementary aid to a conservative interpretation of the estimated burden attributed to dietary risks, taking the consistency of the underlying evidence and the presence of unexplained heterogeneity between studies into account. Low intakes of whole grains and fruits, and high intake of processed meat were leading dietary risks for deaths and DALYs across the Nordic and Baltic countries.

**Implications of all the available evidence**

This study support and extend the conclusions of the NNR 2023 report with evidence about the disease burden that can be attributed to the specific dietary risks in the region. While GBD and NNR used different methods for assessment of optimal intake, current dietary intakes, and their associations to disease outcomes, the conclusions following these two reports are highly aligned. The inclusion of GBD data in NNR exemplifies the usefulness of data from GBD when developing national dietary guidelines. We suggest that GBD data may also be used when dietary guidelines are developed in other countries and regions. The findings in this study may inform priorities for population-level interventions to improve diet.

## Introduction

Unhealthy diet contributes substantially to disease burden in Nordic and Baltic countries,^1^^,^^2^ with implications for public health policies. Knowledge about the magnitude of disease burden attributed to different dietary risk factors in these countries is essential for targeted interventions.^1^ Country-specific comparisons may aid in identifying common and unique dietary challenges. The Global Burden of Diseases, Injuries, and Risk Factors study (GBD) is the world's most comprehensive observational epidemiological study, estimating and comparing the disease burden from 292 causes of death, 375 diseases and injuries, and 88 risk factors, including 15 dietary risks, for 204 countries. GBD, therefore, offers a powerful resource to compare diet-related burden of disease in the Nordic and Baltic countries.

The Nordic countries have a strong tradition for policy collaboration, including collaboration around food policies, with the Nordic Nutrition Recommendations (NNR) as a key example. NNR constitute the scientific basis for national dietary reference values and food-based dietary guidelines for the Nordic and Baltic countries.^3^ Since 1980, the NNR have been updated every 8–10 years, due to an abundance of new scientific studies, and a continuous updating of principles and methodologies for the evaluation of causality in nutritional sciences. The sixth edition, published in 2023,^3^ is a unique regional collaboration involving more than 200 scientists from Denmark, Estonia, Finland, Iceland, Latvia, Lithuania, Norway, and Sweden. A range of systematic reviews and meta-analyses, showing the associations between food intake and health outcomes, are done and published as part of the NNR. NNR does not, however, model and compare diet-related mortality and disease burden in these countries. While GBD and NNR uses somewhat different data-sources and methods for assessment of dietary intake and estimated associations with disease outcomes, results from GBD can aid in the development of NNR guidelines by providing quantitative evidence on the public health impact of unhealthy diet.

The aim of the present study is thus to supplement the NNR by presenting updated data and comparative evidence from GBD 2023 on deaths and disease burden attributed to dietary risks in the Nordic and Baltic countries. This manuscript was produced as part of the GBD Collaborator Network and in accordance with the GBD Protocol.

## Methods

This study is based on results for the year 2023 from GBD 2023^2^ and includes all NNR member countries: Denmark, Estonia, Finland, Greenland (autonomous territory of Denmark), Iceland, Latvia, Lithuania, Norway, and Sweden. The GBD analyses adhere to the Guidelines for Accurate and Transparent Health Estimates Reporting (GATHER) standards (Supplementary Methods Table S1).^4^ The methods for estimating disease burden attributable to risk factors in GBD 2023 are thoroughly described in the causes and risk factors capstone article and its Supplementary Materials.^2^^,^^5^ A short summary of the main steps in estimating disease burden from dietary risks is given below.

GBD classifies risk factors into a four-level hierarchy. The groups on Level 1 includes environmental and occupational risks, behavioural risks, and metabolic risks. Dietary risks are among the Level 2 categories in the behavioural risks group, further broken down into 15 specific dietary risks at Level 3. The 15 dietary risks included in GBD 2023 are defined in Table 1.

**Table 1:** Dietary risk factor definitions and optimal level of exposure as defined in GBD 2023.

Risk type/Dietary factor	Exposure definition	TMREL
**Protective**		
Fruit	Average daily consumption (in grams per day) of fruit, including fresh, frozen, cooked, canned, or dried fruits, excluding fruit juices and salted or pickled fruits	340–350 g/day
Vegetables	Average daily consumption (in grams per day) of vegetables, including fresh, frozen, cooked, canned, or dried vegetables, excluding legumes and salted or pickled vegetables, juices, nuts and seeds, and starchy vegetables such as potatoes or corn	306–372 g/day
Legumes	Average daily consumption (in grams per day) of legumes and pulses, including fresh, frozen, cooked, canned, or dried legumes	100–110 g/day
Whole grains	Average daily consumption (in grams per day) of whole grains (bran, germ, and endosperm in their natural proportion) from breakfast cereals, bread, rice, pasta, biscuits, muffins, tortillas, pancakes, and other sources	160–210 g/day
Nuts and seeds	Average daily consumption (in grams per day) of nuts and seeds, including tree nuts and seeds and peanuts	19–24 g/day
Dietary fibre	Average daily consumption (in grams per day) of fibre from all sources including fruits, vegetables, grains, legumes, and pulses	22–25 g/day
Seafood omega-3 fatty acids	Average daily consumption (in milligrams per day) of eicosapentaenoic acid (EPA) and docosahexaenoic acid (DHA)	470–660 mg/day
Omega-6 Polyunsaturated fatty acids (PUFA)	Average daily consumption (in % daily energy) from omega-6 polyunsaturated fatty acids (PUFA) (specifically linoleic acid, γ-linolenic acid, eicosadienoic acid, dihomo-γ-linolenic acid, arachidonic acid)	9–10% of total daily energy
**Harmful**		
Processed meat	Average daily consumption (in grams per day) of meat preserved by smoking, curing, salting, or addition of chemical preservatives	0 g/day
Sugar-sweetened beverages	Average daily consumption (in grams per day) of beverages with ≥50 kcal per 226.8 serving, including carbonated beverages, sodas, energy drinks, fruit drinks, but excluding 100% fruit and vegetable juices	0 g/day
Sodium	Average 24 h urinary sodium measured (in grams per day)	1–5 g/day
Diet high in trans fatty acids	Average daily consumption (in percent daily energy) of trans fat from industrially produced trans fatty acids mainly from partially hydrogenated vegetable oils	0% of total daily energy
**J-shaped**		
Red meat	Average daily consumption (in grams per day) of unprocessed red meat including pork and bovine meats such as beef, pork, lamb, and goat, but excluding all processed meats, poultry, fish, and eggs	0–200 g/day
**Mixed protective and harmful**		
Milk	Average daily consumption (in grams per day) of dairy milk including non-fat, low-fat, and full-fat milk, but excluding plant-based milks, fermented milk products such as buttermilk, and other dairy products such as cheese	280–340 g/day (males); 500–610 g/day (females)
Dietary calcium	Average daily consumption (in grams per day) of calcium from all sources, including milk, yogurt, and cheese	0.72–0.86 g/day (males); 1.1–1.2 g/day (females)

TMREL = Theoretical minimum risk exposure level.

Risk factor analyses in GBD use a comparative risk assessment framework. The following steps are involved to determine the disease burden of dietary risks: (1) estimating population level dietary intake; (2) calculating the relative risks (RR) of dietary risks on disease outcomes; (3) determining the theoretical minimum risk exposure level (TMREL); and (4) estimating the risk-attributable disease burden for each dietary risk and calculated by year, location, sex, and age group. With GBD 2021, the Burden of Proof Function Methods (BPRF) to assess the strength of evidence for specific risk–outcome relationships was introduced. Dietary risk modelling in GBD is done for adult populations above age 25.

### Dietary intake estimation

Dietary intake in GBD was modelled using multiple data sources, including national and subnational nutrition surveys, household budget surveys, accounts of national sales from the Euromonitor, and food availability data from the United Nations Food and Agriculture Organisation's supply and utilisation accounts. The dietary data sources for the Nordic and Baltic countries included in GBD 2023 are listed in the GBD 2023 data source tool Global Health Data Exchange (GHDx).^6^ For locations with missing or sparse data on certain risk factors, the exposure level of the GBD region to which the location belongs, based on geographical and epidemiological proximity, is used as a prior in the model estimating exposure. The GBD regions are as follows: Western Europe for the Nordics, Eastern Europe for the Baltic countries, and high-income North-America for Greenland. The gold-standard for dietary intake was 24-h dietary recall surveys for all dietary risks except sodium, where 24-h urinary sodium was considered the gold-standard. Alternative measures of dietary intake were converted to the gold-standard definition using meta-regression or network meta-regression, accounting for biases associated with the data, like food wastage in sales data and underreporting of food intake in self-reported questionnaires. Detailed methods are provided in GBD 2023 capstone Supplementary Material.^2^

### Theoretical minimum exposure level

Theoretical minimum risk exposure level (TMREL) is the counterfactual exposure level that minimises health risks at a population level, identified through epidemiological evidence.^2^^,^^5^ For harmful dietary risks, where exposure can theoretically be eliminated, TMREL was set to zero. For protective dietary risks, TMREL was defined as the lowest intake level associated with reduced mortality across disease outcomes. Mixed risks, like calcium and milk, followed the same approach, but incorporated the 15th percentile of the upper bound for harmful risk-outcome pairs. For J-shaped risks (red meat), TMRELs were derived from identifying the intake level that minimised the mean relative risk curve, using weighted all-cause mortality curves. Sodium intake was assigned a TMREL uncertainty range of 1–5 g per day reflecting the lack of consensus on the optimal level.^5^ In GBD 2023, trans fats were limited to industrial sources, with the TMREL set to zero. Detailed definitions for each risk are outlined in Table 1.

### Relative risks and strength of evidence

GBD 2023 analysed 44 direct relationships between dietary risks and disease outcomes using evidence from randomised-controlled trials and cohort studies (Supplementary Methods Table S2). Consistent with the World Cancer Research Funds's criteria,^7^ risk-outcome pairs were included if there was convincing or probable evidence of an association.^1^ Risk curves were modelled using the meta-regression, Bayesian, regularised, trimmed (MR-BRT) tool, identifying monotonic risks (consistently harmful or protective) and allows for non-linear dose–response relationships, while adjusting for important covariates.^5^ Relative risks were calculated across dietary intake levels for age groups 25–95+ years. To assess the strength of evidence for each association, GBD applies BPRF, which considers effects size, study heterogeneity, and uncertainty, rating associations on a one-to-five–star scale (no evidence to very strong evidence).^8^ The risk-outcome pairs and their BPRF star ratings are listed in Supplementary Methods Table S2.

In order to appropriately aggregate risk attributable burden up the risk factor hierarchy (to clusters of risk factors or all risks), GBD also models indirect risk–outcome relationships through mediation. Many dietary risks are associated with disease outcomes mediated through metabolic risks, like diets high in sodium on hypertensive heart disease mediated by high systolic blood pressure or diets low in whole grain on ischemic heart disease mediated through high LDL cholesterol. Details on mediation modelling for dietary risks are available in the GBD 2023 Risk Factor capstone Supplementary Material.^5^

### Disease burden from dietary risks

GBD uses several measures to describe disease burden,^2^^,^^9^^,^^10^ but this study focuses on deaths and disability-adjusted life-years (DALY). DALY is the summary measure of disease burden, including total health loss in a population due to years of life lost (YLL) and years lived with disability (YLD). The proportion of deaths and DALYs attributed to each dietary risk (the population-attributable fraction, PAF), is calculated using GBD comparative risk assessment approach.^2^ PAF estimates the proportional reduction in population disease burden if population exposure were set to the TMREL. Risk-specific disease burdens were determined by multiplying the PAF by total disease-specific deaths and DALYs for each location.

### Statistical analyses

All estimates presented in this study were provided by the GBD dietary risk factor team or retrieved from the online GBD Results Tool.^11^ GBD organises causes into a four-level hierarchy, ranging from all-cause aggregates (level 0) to increasing details. This study presents Level 2 disease groups (e.g., cardiovascular diseases and neoplasms) and Level 3 specific causes (e.g., ischaemic heart disease or diabetes mellitus). Dietary intake is reported in grams or milligrams per day, depending on the nutrient or food group, or percentage of daily energy consumption. The burden of overall and individual dietary risks is described using the number (all ages) and age-standardised rates (ASR) per 100,000 inhabitants of deaths and DALYs attributed to each risk. Age-standardisation follows the standard world population developed for GBD 2023. All estimates include 95% uncertainty intervals (UI)], reflecting uncertainty from measurement bias, stochastic variation, and model specification. UIs were calculated using 1000 draws from the posterior distribution, with point estimates derived from the mean, and the 2.5th and 97.5th percentiles defining UI bounds.

### Role of the funding source

The funders of the study had no role in study design, data collection, data analysis, data interpretation, preparing the report, or decision to publish.

## Results

### Dietary intake

**Fig. 1:**
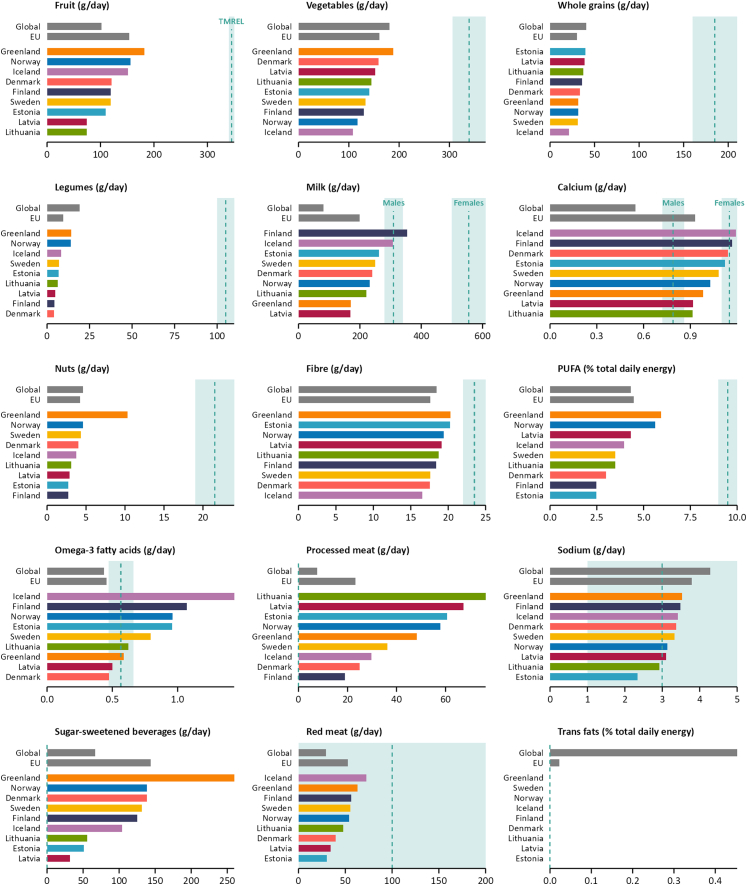
Intake of 15 dietary risks among adults of both sexes aged 25 years or older in the Nordic and Baltic countries, 2023, with theoretical minimum exposure level (TMREL) shown as a range (light green panel) and midpoint (dashed line).

Across the Nordic and Baltic countries, the intake of fruit, vegetables, whole grains, legumes, nuts, fibre, and PUFA, all defined as protective dietary risks, was below the TMREL range, whilst the intake of processed meat and sugar-sweetened beverages, where the TMREL was defined as zero intake, was above the TMREL (Fig. 1). For sodium and the J-shaped dietary risk red meat, the intake was within the TMREL range. A zero percent intake of trans fats was assumed for all countries, as they have now introduced bans on industrially produced trans fats.

### All cause and cause specific disease burden attributable to all dietary risks

**Fig. 2:**
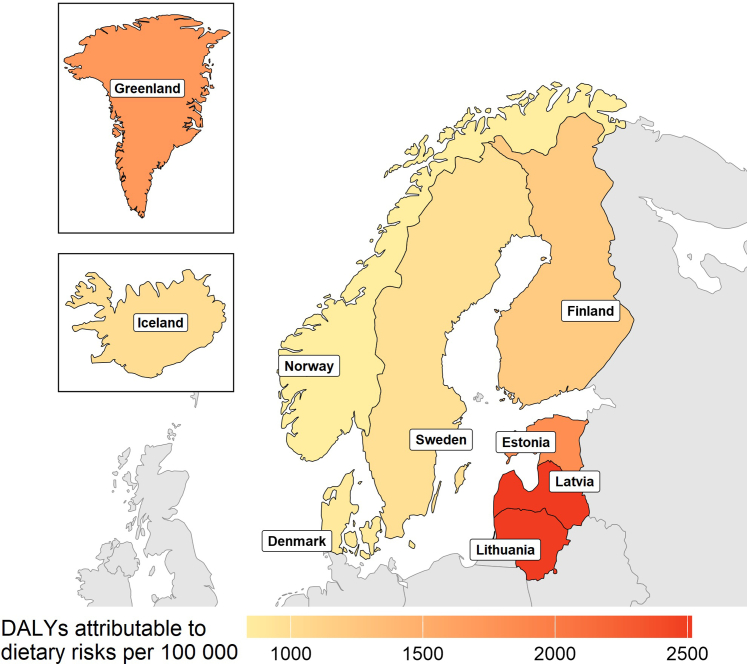
Age-standardised rates of disability-adjusted life-years (DALYs) attributable to dietary risks per 100,000 inhabitants in the Nordic and Baltic countries, 2023.

In a total population of 34,064,020 individuals across all Nordic and Baltic countries, a total of 38,450 deaths (95% UI 10,749–59,386) and 735,284 DALYs (242,417–1,06,638) were attributed to dietary risks in 2023. ASR of death attributable to dietary risks ranged from 33.7 (15.5–49.3) in Norway, to 118.5 (28.6–183.5) in Lithuania, while ASRs of DALYs ranged from 837.4 (433.9–1178.7) in Norway to 2514.8 (1000.4–3581.9) in Latvia (Table 2, Fig. 2 and Supplementary Results Figure S1). The proportion of each country's overall number of deaths attributable to dietary risks ranged from 8.9% in Denmark (2.2–13.8) and Norway (4.0–13.4) to 19.8% (4.5–31.2) in Lithuania, and DALYs from 5.6% (2.8–8.1) in Norway to 12.2% (3.5–18.3) in Lithuania (Table 2).

**Table 2:** Numbers, age-standardised rates (ASR) per 100,000 inhabitants, and proportions of deaths and disability-adjusted life-years (DALYs) attributed to dietary risk factors for the Nordic and Baltic countries, 2023.

	Count (95% UI)	ASR per 100,000 (95% UI)	Proportion of total (%) (95% UI)
**Deaths**			
Latvia	4910 (1705–7359)	111.4 (40.2–164.4)	17.6 (6.2–26.5)
Lithuania	7665 (1743–12,003)	118.5 (28.6–183.5)	19.8 (4.5–31.2)
Estonia	2968 (1463–4246)	91.3 (43,2–129.7)	18.2 (9.0–26.0)
Greenland	44 (22–65)	84.4 (42.5–126.3)	8.6 (4.3–12.6)
Finland	8221 (1093–13 097)	54.6 (7.6–85.3)	13.4 (1.8–21.4)
Sweden	10,828 (3002–16 779)	41.7 (12.1–63.6)	11.5 (3.2–17.8)
Iceland	281 (76–446)	43.3 (12.1–67.9)	10.9 (3.0–17.3)
Denmark	5234 (1309–8036)	38.9 (9.2–59.0)	8.9 (2.2–13.8)
Norway	3890 (1748–5765)	33.7 (15.5–49.3)	8.9 (4.0–13.4)
Nordic and Baltic	38,450 (10,749–59,386)	50 (15–76)	n.a.
**DALYs**			
Latvia	96,395 (36,619–140,543)	2514.8 (1000.4–3581.9)	11.6 (4.5–16.9)
Lithuania	145,366 (40,482–215,064)	2508.3 (743.3–3653.0)	12.2 (3.5–18.3)
Estonia	51,487 (23,498–71,533)	1827.9 (794.2–2553.8)	10.4 (4.9–14.4)
Greenland	1113 (582–1591)	1723.5 (895.8–2531.3)	5.1 (2.7–7.3)
Finland	148,459 (25,691–224,907)	1202.0 (235.6–1769.0)	8.1 (1.4–12.3)
Sweden	208,884 (68,375–305,496)	989.8 (345.3–1400.5)	6.9 (2.3–10.3)
Iceland	5845 (1921–8634)	991.8 (338.2–1455.9)	6.0 (2.2–9.1)
Denmark	103,071 (27,332–151,760)	882.0 (243.6–1279.4)	5.7 (1.5–8.6)
Norway	83,249 (41,180–118,006)	837.4 (433.9–1178.7)	5.6 (2.8–8.1)
Nordic and Baltic	735,284 (242,417–1,06,638)	1144 (406–1617)	n.a.

95% uncertainty intervals shown in parentheses.

**Fig. 3:**
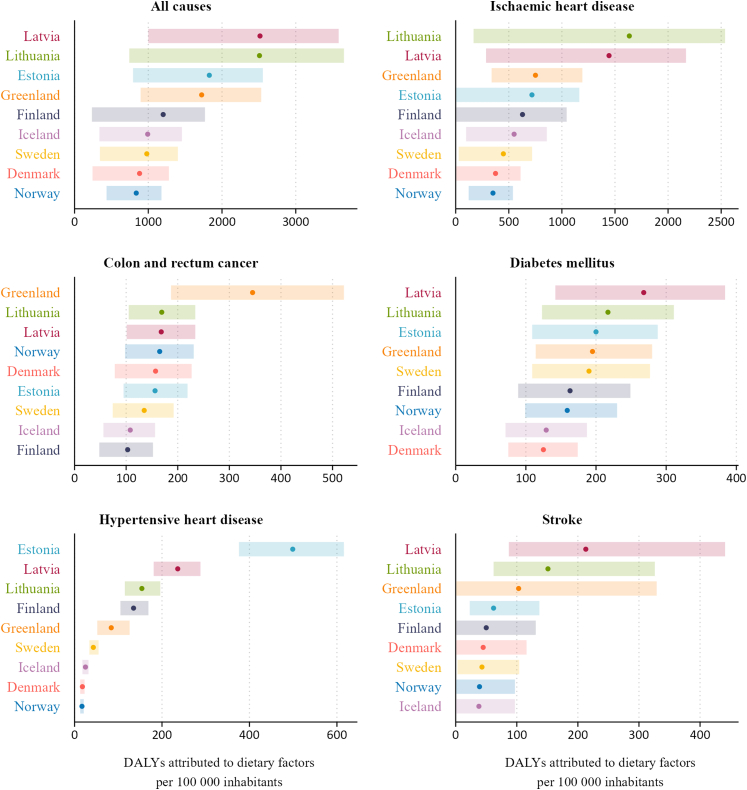
Disability-adjusted life-years (DALYs) attributed to all dietary risks for the leading diet-related level 3 causes and all causes in the Nordic and Baltic countries, 2023. Age-standardised rates for per 100,000 inhabitants. Background shaded areas represent 95% uncertainty intervals.

For all Nordic and Baltic countries combined, dietary risks accounted for 24.9% of the total DALYs from cardiovascular diseases (5.05–37.63), 29.6% of the DALYs from diabetes and kidney diseases (18.6–40.0), and 7.8% of the DALYs from neoplasms (2.9–12.1) (data not shown). The main GBD Level 3 causes of DALYs attributable to dietary risks were ischaemic heart disease, diabetes mellitus, colorectal cancer, hypertensive heart disease and stroke. While the ASR point estimates of DALYs may indicate some cause-specific differences between countries, for instance, a higher burden for ischaemic heart disease and stroke in the Baltic versus the Nordic countries, the wide and overlapping uncertainty intervals limit the strength of these comparisons, except from significantly higher diet-attributed disease burden for hypertensive heart disease in Estonia compared to the other countries (Fig. 3).

### Disease specific burden from individual dietary risks

**Fig. 4:**
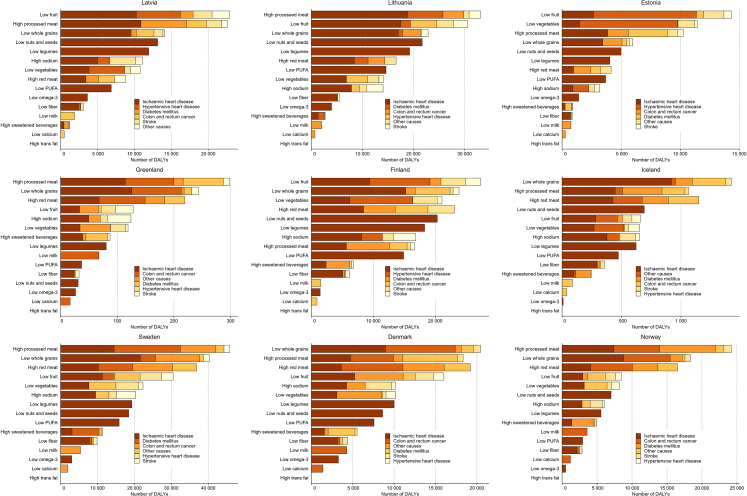
Number of disability-adjusted life-years (DALYs) attributable to individual dietary risks in the Nordic and Baltic countries in 2023, by level 3 causes. Note: Trans-fat appears with a zero estimate because industrial trans-fat has been banned in the included countries, and population intake is assumed to be zero.

When assessed at the country level, the leading dietary risks for DALYs were broadly similar across the Nordic and Baltic countries, with some variation in ranking. High intake of processed meat, low intake of fruit, and low intake of whole grains consistently ranked among the top five dietary risks in nearly all countries (Fig. 4; Supplementary Table S1). High intake of red meat, low intake of nuts and seeds, and low intake of vegetables also frequently ranked among the top five across countries. The highest count of DALYs attributed to low intake of fruit was found for Lithuania 30,644.9 (11,151.0–49,638.4), while the ASRs for DALYs ranged from 87.9 (31.8–161.1) in Norway to 616.3 (248.3–947.4) in Latvia. For low intake of whole grains and high intake of processed meat, Sweden had the highest count of DALYs (40,321.7 (18,327.3–65,955.5); and 45,844.9 (23,465.3–69,502.3) respectively), while Lithuania (401.0 (179.0–652.7) had more than double the ASR of DALYs than Denmark (176.3 (75.1–273.2) for low intake of whole grains, and Latvia (613.9 (174.9–994.9)) had more than four times the ASR of Finland (155.5 (74.1–280.5)) due to high intake of processed meat (Fig. 4, Supplementary Results Figures S2 and Table S1).

## Discussion

Our analyses indicate suboptimal intake of most of the 15 dietary risks in GBD 2023 across the Nordic and Baltic countries, resulting in a substantial disease burden in these countries being attributed to dietary risks. The dietary risks with the highest impact across countries included low intakes of fruits, whole grains, vegetables, and nuts and seeds, and high intakes of processed meat, and red meat. These risks increased disease burden due to ischaemic heart disease, diabetes mellitus, hypertensive heart disease, stroke, and colon and rectum cancer. The impact of dietary risks varied between countries, with a generally higher burden in the Baltic countries and Greenland compared to the Nordic countries. These results highlight the critical role of unhealthy diet in disease burden, necessitating targeted strategies to improve diet across the Nordic and Baltic region.

GBD's quantification of deaths and disease burden due to dietary risks provides a compelling case for the public health potential in targeting unhealthy diet. This gives important background evidence for diet related guidelines and policy recommendations, such as the NNR.^3^ While GBD and NNR used different methods for assessment of optimal intake, current dietary intakes and their associations to disease outcomes (see the Supplementary Methods page 2 for details), it is notable that their conclusions are highly aligned. Both sources emphasise the importance of focussing on increasing the intake of whole grains, fruits, and vegetables, while reducing the intake of unprocessed and processed red meat, and sodium to improve public health in the region. The intake recommendations in NNR are largely in line with the TMRELs for the most important food groups, i.e. fruits and vegetables, unprocessed and processed red meat, milk and dairy products, and nuts and seeds. NNR did not give a concrete recommendation of intake of legumes and pulses, but some of the national guidelines, like Denmark,^12^ have recommendations that are similar to the TMREL of GBD. The largest discrepancy between NNR and GBD was for cereals/whole grains, where the TMREL from GBD is 160–210 g per day, while NNR recommends a daily intake of 90 g per day but emphasise that there are likely benefits with further increasing the intake. When comparing GBDs TMRELs and NNRs recommended intake, it's important to note that GBD's TMRELs are not intended as standalone guidelines but serve to inform guideline development. Guidelines often incorporate a “safety factor” and draw on multiple data sources, while GBD's diet-related TMRELs are based on RCTs and cohort studies. Additionally, TMRELs are inherently global, whereas guidelines may account for regional intake patterns (e.g., Nordic diets) and can vary across locations.

The GBD results highlights both shared and unique challenges between the countries. The higher diet-related disease burden in the Baltic countries (Estonia, Lithuania, and Latvia) and Greenland compared to the Nordic countries is both due to more suboptimal dietary intake and a generally higher disease burden. The latter is reflected in substantially lower life expectancies, ranging from 75.8 to 78.7 years in the Baltic countries versus 81.5 to 83.3 years in the Nordic countries (excluding Greenland),^9^ and higher age-standardised disease burden due non-communicable diseases,^2^ despite the improvements seen in the past two decades (Supplementary Results Figures S3–S5).

While the health effects of foods are generally consistent across populations, local contexts, including public health challenges and population age structure, food availability, traditions, and culture, and socioeconomic factors play a crucial role.^13^^,^^14^ Important within-country disparities in diet related disease burden also exist, driven by socioeconomic factors. Even in European welfare states, constraints on access to nutritious diets for vulnerable populations still exist, driven by purchasing power.^15^^,^^16^ Obesity, which is a common consequence of unhealthy diet and a strong driver for other metabolic risks, is also more prevalent among socially disadvantaged groups.^17^ The differences in diet-related diseases across the Nordic and Baltic countries can be attributed to a combination of these factors and this knowledge must inform policy recommendations.

While GBD has shown the public health impact of the unhealthy diet, and NNR has provided evidence-based guidelines for optimal intake, it is up to the policy makers and the food production system to implement effective interventions. It has been emphasised that a comprehensive policy approach involving and focusing on coherence across a range of sectors, including the food production sector, is necessary to enhance adherence to national dietary guidelines beyond education and awareness campaigns.^3^^,^^13^^,^^18^^,^^19^ Suggested interventions targeted towards the consumers include front-of-package labelling, subsidies for healthy foods, taxes or bans on unhealthy foods (such as the ban on trans fat), marketing regulations, healthcare professional involvement, and national surveillance.^18^^,^^20^^,^^21^ Policymakers must also address barriers such as poor coordination between policy sectors, economic policies, limited access to healthy foods, cultural and social influences, and behavioural preferences.^13^^,^^18^^,^^19^

In addition to the public health aspect, the impact from food systems on the environment and climate should also be taken into consideration when developing policies. The current agriculture and food systems contribute to over one-third of greenhouse gas emissions and more than two-thirds of biodiversity loss and deforestation.^22^ In addition, one-third of all food produced is lost or wasted.^22^^,^^23^ Thus, transforming governmental subsidies and food systems to those that supports a healthy and sustainable diet, is essential to address global environmental, food security, and public health crises.^3^^,^^14^^,^^24^

Decisions about whether to include a given risk-outcome pair in GBD is based on analytic feasibility, evidence of a causal association, and priorities, such as the potential impact on disease burden or policy. The list of included pairs in GBD evolves constantly, and therefore, cannot be regarded as complete in terms of all potential outcomes that can be associated with dietary risks. Each step in the GBD's risk methodology, including determining risk exposure, TMREL, cause-specific mortality and morbidity, risk–outcome relationships, and attributable burden, involves uncertainty. Although uncertainty intervals address much of this, underlying assumptions regarding causality and methodology must be considered. Additionally, most dietary risk-disease outcome associations are weak, as reflected in one- or two-star rating in GBD's BPRF models. Consequently, the study's estimates involve substantial uncertainty and should be interpreted with caution.

Causal inference is inherently challenging in observational research, particularly in diet research, due to confounding factors and exposure misclassification. GBD mitigates these biases by systematically reviewing available evidence and excluding case–control or cross-sectional studies from meta-analysis of the dietary risks, reducing the potential for associations to result from reverse causation. The BPRF method explicitly considers the consistency of evidence and accounts for unexplained heterogeneity. Studies are carefully assessed for confounder control, with incomplete controls flagged and incorporated as covariates in the meta-regression model to adjust the pooled risk estimates. Robust methods to detect and trim outliers, estimate between-study heterogeneity, adjust for the number of studies, and adjust for study design, further enhance reliability. However, as with all observational research, residual confounding cannot be ruled out.^2^^,^^8^

While the median risk functions identified through the BPRF methodology suggested protective, harmful, or j-shaped associations for the 44 dietary risk-outcome pairs included in GBD, 11 pairs received one-star ratings (possibly no association), 27 were rated as two-star (weak evidence), and 6 were rated as three-star risk–outcome relationships (moderate evidence). None received four (strong evidence) or five-stars (very strong evidence) (Supplementary Methods Table S2). The low star ratings, which is based on risk magnitude and between-study heterogeneity, reflects the large uncertainty in the evidence for many dietary risk–outcome relationships, such as outcomes associated with intake of unprocessed red meat.^25^ For risk factors with high uncertainty, there is an urgent need for high-quality large-scale longitudinal studies with reliable measures of exposure (e.g., dietary intake) to better inform future recommendations and policies. Further, the BPRF provides a conservative estimate of the association between risk and outcome, as it is based on the risk level consistent with available data that is closest to the null hypothesis.^8^ Accordingly, dietary recommendations like NNR should interpret these findings cautiously. However, the precautionary principle supports public health recommendations even for lower-rated risks,^2^ particularly when exposure is widespread or linked to several disease outcomes, as is the case with most dietary risks.

GBD uses various sources to estimate dietary risk factor intakes. Although 24-h dietary recall surveys are the gold standard for all dietary risks except sodium, they are unavailable in many regions. GBD relies heavily on data obtained from the United Nations Food and Agriculture Organisation's supply and utilisation accounts, covering 245 countries and territories.^26^ Food availability data must be adjusted for factors like wastage and modelled for intake differences between sexes and age-groups. Consequently, GBD intake estimates may differ from those obtained from national dietary surveys, like those informing the NNR. While the GBD estimation framework is highly valuable for its ability to provide comparable dietary intake estimates across diverse regions, it may diverge from data collected through national dietary surveys due to methodological differences. For instance, the unexpectedly high intake levels of fruits, vegetables, and whole grains observed in Greenland and the notably low salt intake in the Baltic countries illustrate potential discrepancies stemming from the granularity of GBD, as well as the Bayesian framework used by GBD, which integrates data from multiple sources, including those from other neighbouring countries where local data are unavailable. These differences highlight the need for careful interpretation of such data, particularly when national surveys are available to provide a more nuanced picture of actual dietary intake levels.

Since GBD is included as background for the latest NNR edition,^3^ it is important to consider methodological differences between these two sources. The intake estimates used in NNR^3^^,^^27^, ^28^, ^29^, ^30^ and GBD,^2^ are similar, for most food groups and nutrients, but not identical. These differences are discussed in Supplementary Methods page 2. GBD calculates the TMREL based on all-cause mortality, which may not accurately capture specific disease–risk relationships. The TMREL is based on the intake associated with the lowest overall risk, which can be complicated due to opposing effects on various diseases. Further, the TMRELs and RRs are only based on the total relationships between dietary risk and disease outcomes, and unaffected by the separate mediation analysis. Finally, GBD only estimates dietary risks for those over 25, excluding younger populations. GBD is constantly evolving, with new data and evidence impacting results and interpretations. While this dynamic nature of GBD is a major strength, it can make comparisons between iterations challenging. Due to the data updates and methodological improvements following each round of GBD, the latest iteration supersedes all previous rounds.

### Conclusion

A substantial disease burden can be attributed to dietary risks in the Nordic and Baltic countries, especially diets high in processed meats, and low in fruits and whole grains. These findings support and extend the conclusion of the NNR report. Successful implementation of policies targeting dietary risks could substantially reduce disease burden in all Nordic and Baltic countries. The results should be interpreted with caution, due to weak evidence supporting most dietary risk–disease outcome associations.

## Contributors

Please see Supplementary Material (p. 17) for more detailed information about individual author contributions to the research, divided into the following categories: providing data or critical feedback on data sources; developing methods or computational machinery; providing critical feedback on methods or results; drafting the manuscript or revising it critically for important intellectual content; and managing the estimation or publications process. All data were verified by members of the core writing team (AKS. Knudsen, C. Baravelli and C. Madsen), and by analysts at IHME (M. Brauer and D. Haile). All other authors had access to and reviewed estimates as part of the research evaluation process, which includes additional stages of formal review. The corresponding author (AKS. Knudsen) was responsible for the decision to submit the manuscript.

## Data sharing statement

To download the data used in these analyses, please visit the Global Health Data Exchange GBD 2023 website at https://vizhub.healthdata.org/gbd-results/. Data for the Baltic and Nordic countries combined can be obtained by contacting the first author.

## Editor note

The Lancet Group takes a neutral position with respect to territorial claims in published maps and institutional affiliations.

## Declaration of interests

M Kivimäki reports support for their participation in the current manuscript from Wellcome Trust, UK (221854/Z/20/Z), National Institute on Aging (NIH), US (R01AG056477), Medical Research Council, UK (MR/R024227/1, MR/Y014154/1), and Academy of Finland (350426). All other authors had nothing to declare.

## Appendix A Supplementary data

Supplementary Figures and Tables

Study Group
