# Supplementary material for: The burden of dietary risk factors in the Nordic and Baltic countries: a systematic analysis for the Global Burden of Disease Study 2023
Source: Lancet Reg Health Eur. 2025 Nov 25;61:101543. doi: 10.1016/j.lanepe.2025.101543 (PMC12686888; doi:10.1016/j.lanepe.2025.101543)
Supplement: Supplementary Figures and Tables [file mmc1.docx]

Supplementary materials to: The burden of dietary risk factors in the Nordic and Baltic countries: A systematic analysis for the Global Burden of Disease Study 2023

# Supplementary methods

## Difference in methodologies used by GBD and NNR

Since GBD is included as background for the latest NNR edition, it is important to consider methodological differences between these two sources. The intake estimates used in NNR and GBD are similar, but not identical for most food groups and nutrients. NNR relies on smaller, high-quality datasets, while GBD uses a larger dataset that allows computation of missing values. Consequently, GBD’s intake estimation is partly data-driven, which may explain some differences between GBDs intake estimates and those used in the NNR.^1^ The methodology for assessing relationships between dietary risks and disease outcomes differ between GBD and NNR. Unlike GBD, NNR applies stricter inclusion and exclusion criteria for systematic reviews, emphasising non-bias and high-quality evidence.^2-4^ Therefore, only “strong causal relationships” defined by NNR as used as the basis for its recommendations, whereas GBD includes several other relationships in its risk functions. Another difference in methodology is the novel Burden of Proof Risk Function Models (BPRF) in GBD which gives more conservative risk estimates that the 95% confidence intervals typically used in classical meta-analyses and NNR. Moreover, GBD incorporates additional modelling techniques. For example, GBD employs Meta-Regression - Bayesian Regularised Trimmed (MR-BRT), the Bayesian meta-regression tool, when calculating dose-response curves for specific dietary risk-disease endpoint pairs. This uses a Bayesian framework to integrate prior knowledge, trims outliers to reduce outlier bias, adjusts for systematic errors (e.g., food wastage, underreporting), includes covariates, and allows for non-linear dose-response relationships using splines (typically increases steeply at lower levels of exposure and then plateau at higher levels, whereas traditional methods may just be log-linear). These features ensure a more reliable risk assessment, especially for sparse or noisy data.

Additionally, the TMRELs in the present analyses are similar but not identical to NNR recommendations. Another distinction is the number of dietary risks and health outcomes assessed. GBD focuses on 15 dietary risks, while NNR assesses 18 food groups, 35 nutrients, and meal and dietary patterns.^5^ Also, while alcohol use is included in NNR, high alcohol use is not analysed as part of the dietary risks in GBD, but as its own Level 2 behavioural risk category.

### References

1. Lemming EW, Pitsi T. The Nordic Nutrition Recommendations 2022 - food consumption and nutrient intake in the adult population of the Nordic and Baltic countries. *Food Nutr Res* 2022; **66**.

2. Arnesen EK, Christensen JJ, Andersen R, et al. The Nordic Nutrition Recommendations 2022 - structure and rationale of qualified systematic reviews. *Food Nutr Res* 2020; **64**.

3. Arnesen EK, Christensen JJ, Andersen R, et al. The Nordic Nutrition Recommendations 2022 - handbook for qualified systematic reviews. *Food Nutr Res* 2020; **64**.

4. Christensen JJ, Arnesen EK, Andersen R, et al. The Nordic Nutrition Recommendations 2022 - principles and methodologies. *Food Nutr Res* 2020; **64**.

5. Blomhoff R, Andersen R, Arnesen EK, et al. Nordic Nutrition Recommendations 2023. Copenhagen: Nordic Council of Ministers, 2023.

**Table S1. Checklist for Guidelines of Accurate and Transparent Health Estimates Reporting (GATHER)**

**
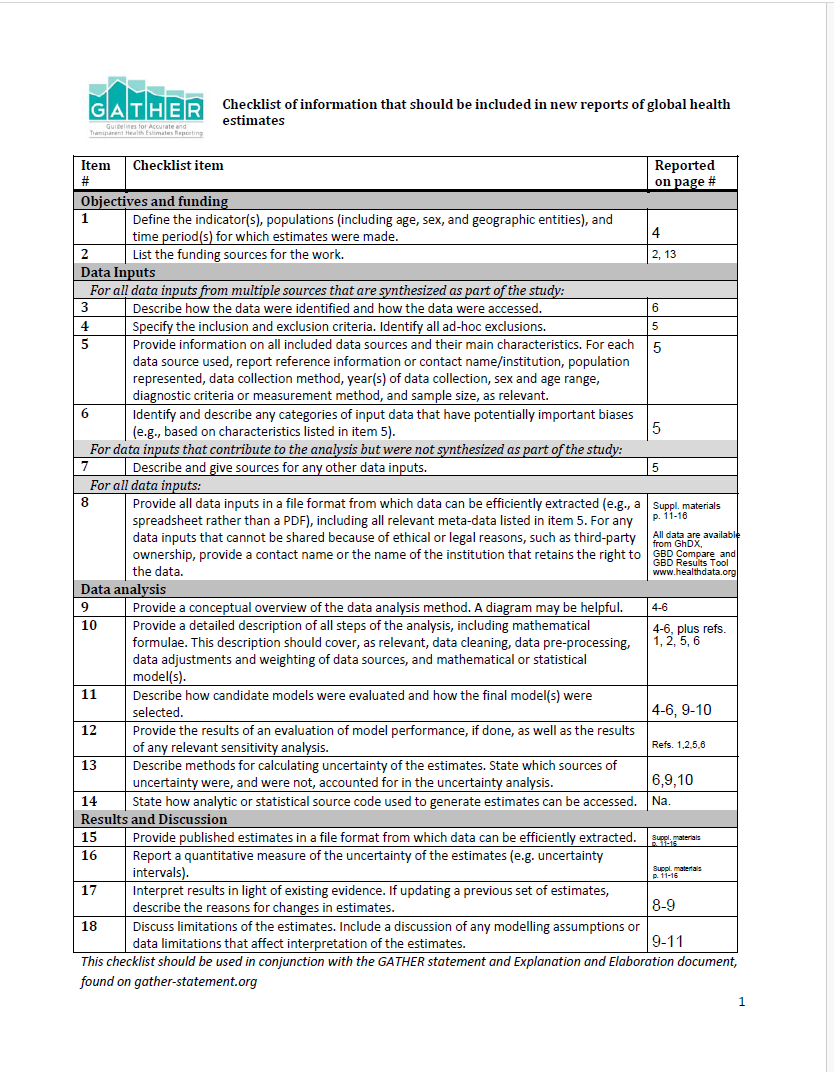
**

**Table S2. Dietary risk and disease outcome relationships analysed in the GBD 2023 Burden of Proof tool (BPRF), with the resulting star rating*.**

| **Risk** | **Outcome** | **Star rating** |
| --- | --- | --- |
| Diet high in processed meat | Diabetes mellitus type 2 | 2 |
|  | Ischaemic heart disease | 1 |
|  | Colon and rectum cancer | 2 |
| Diet high in red meat | Colon and rectum cancer | 2 |
|  | Breast cancer | 2 |
|  | Ischaemic heart disease | 2 |
|  | Diabetes mellitus type 2 | 2 |
|  | Ischaemic stroke | 1 |
|  | Intracerebral haemorrhage | 1 |
|  | Subarachnoid haemorrhage | 1 |
| Diet high in sodium | Stomach cancer | 3 |
| Diet high in sugar-sweetened beverages | Diabetes mellitus type 2 | 2 |
|  | Ischaemic heart disease | 2 |
| Diet high in trans fatty acids | Ischaemic heart disease | 2 |
| Diet low in calcium | Colon and rectum cancer | 3 |
|  | Prostate cancer | 2 |
| Diet low in fibre | Colon and rectum cancer | 2 |
|  | Ischaemic heart disease | 2 |
|  | Diabetes mellitus type 2 | 2 |
|  | Ischaemic stroke | 2 |
|  | Intracerebral haemorrhage | 1 |
|  | Subarachnoid haemorrhage | 1 |
| Diet low in fruits | Tracheal, bronchus, and lung cancer | 3 |
|  | Ischaemic stroke | 3 |
|  | Ischaemic heart disease | 2 |
|  | Diabetes mellitus type 2 | 2 |
|  | Intracerebral haemorrhage | 2 |
|  | Subarachnoid haemorrhage | 2 |
| Diet low in legumes | Ischaemic heart disease | 1 |
| Diet low in milk | Colon and rectum cancer | 2 |
|  | Prostate cancer | 1 |
| Diet low in nuts and seeds | Ischaemic heart disease | 2 |
| Diet low in omega-6 polyunsaturated fatty acids | Ischaemic heart disease | 1 |
| Diet low in seafood omega-3 fatty acids | Ischaemic heart disease | 2 |
| Diet low in vegetables | Ischaemic stroke | 3 |
|  | Ischaemic heart disease | 2 |
|  | Intracerebral haemorrhage | 2 |
|  | Subarachnoid haemorrhage | 2 |
|  | Oesophageal cancer | 2 |
|  | Diabetes mellitus type 2 | 1 |
| Diet low in whole grains | Ischaemic heart disease | 3 |
|  | Diabetes mellitus type 2 | 2 |
|  | Colon and rectum cancer | 2 |
|  | Ischaemic stroke | 1 |

*The star rating reflects the strength of evidence of an association between risk and outcome, using a one to five star-rating system to rank each pair based on the magnitude and consistency of risk shown by studies to date, with higher star ratings signifying greater strength of evidence: one-star rating=possibly no association, two-star rating =weak evidence of association, three-star rating=moderate evidence of association, four-star rating=strong evidence of association, five-star rating =very strong evidence of association. Note that further details, including the specific references used to obtain the ratings and relative risk curves, can be found at https://vizhub.healthdata.org/burden-of-proof.


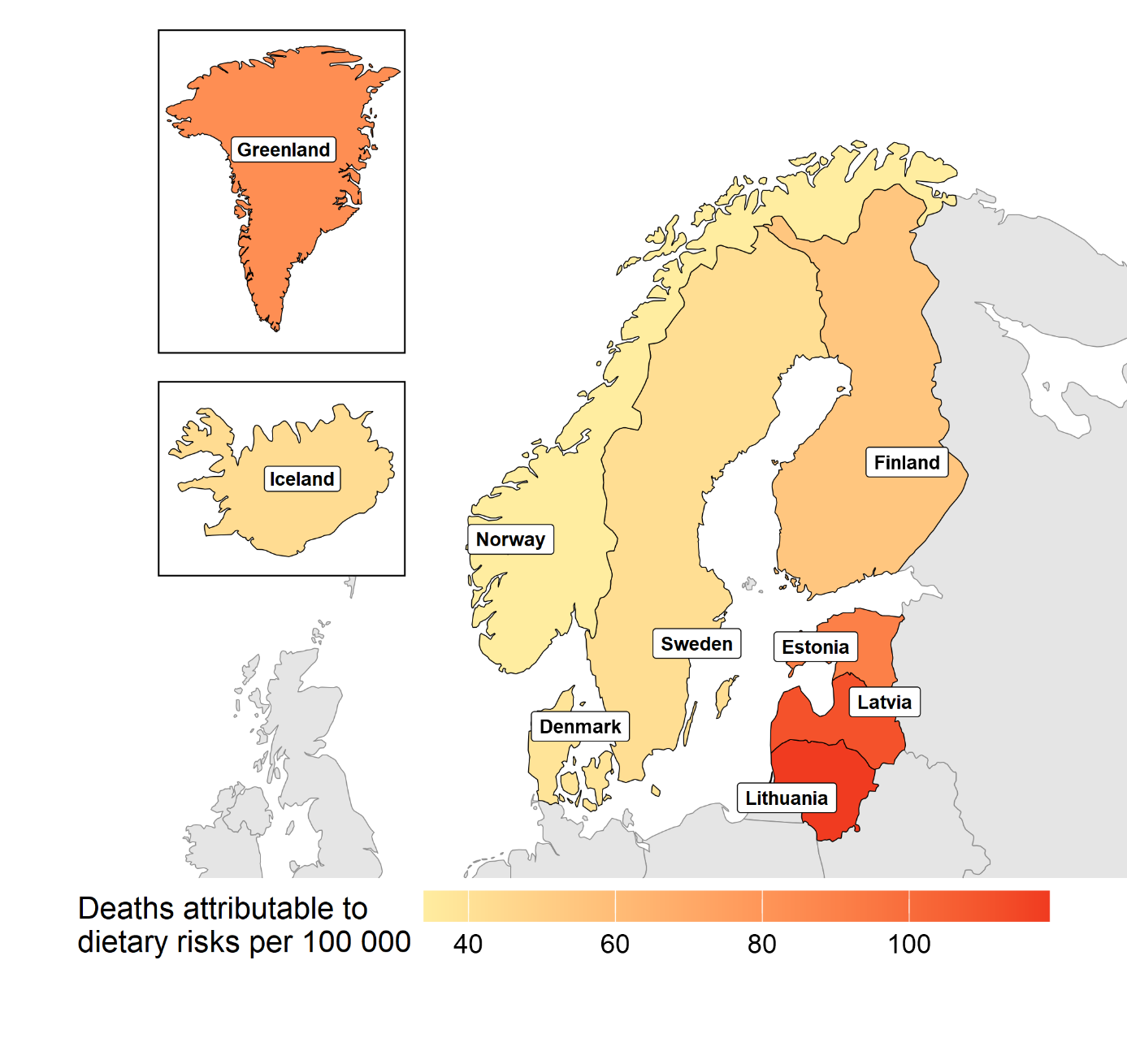
Figure S1. Age-standardised rates (ASR) of deaths attributable to dietary risks per 100 000 inhabitants in the Nordic and Baltic countries, 2023.


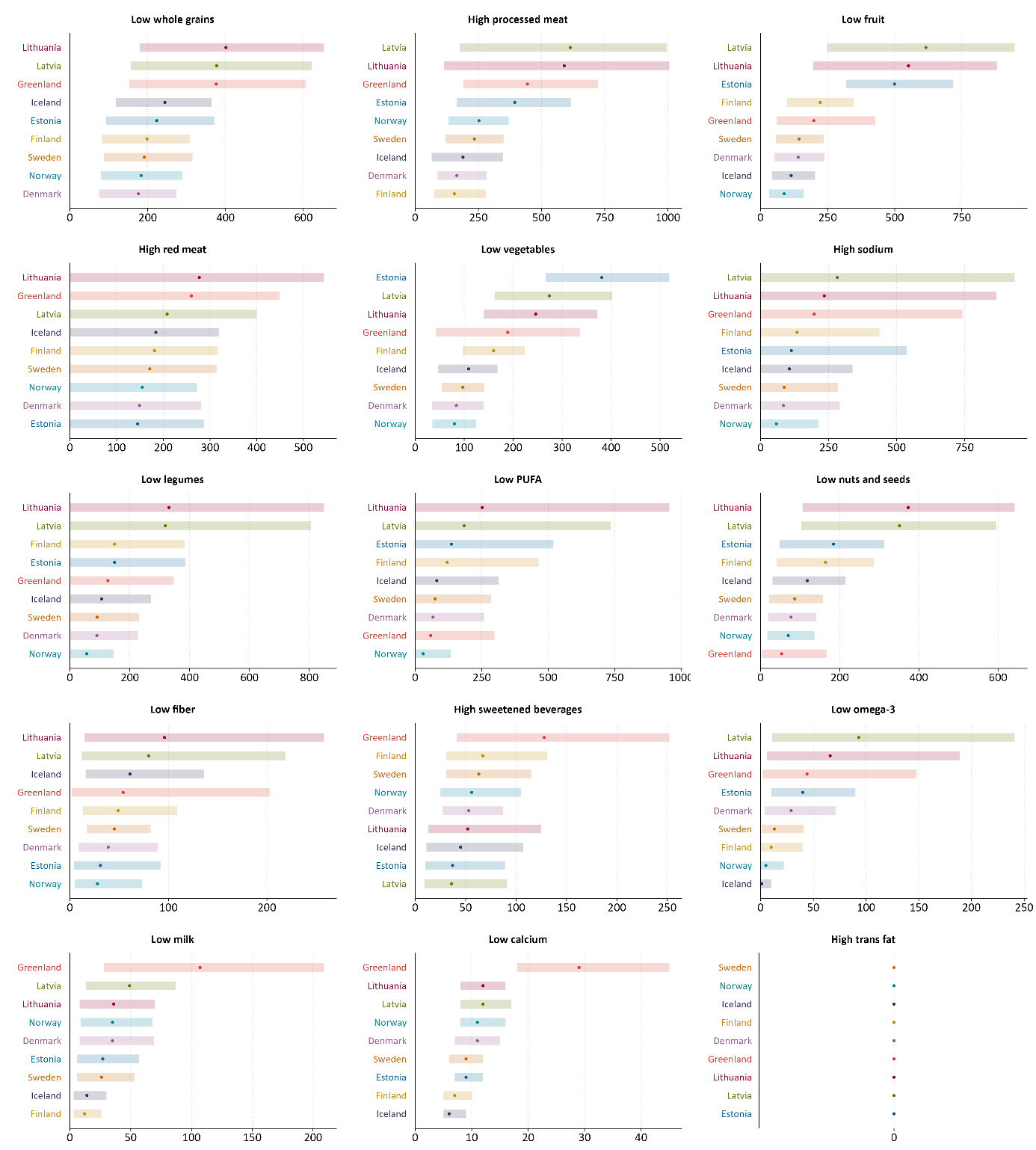


Figure S2. Age-standardised rates (ASR) of disability-adjusted life-years (DALYs) attributable to individual dietary risks per 100 000 inhabitants in the Nordic and Baltic countries. Background bands represent 95% uncertainty intervals. Results from GBD 2023.


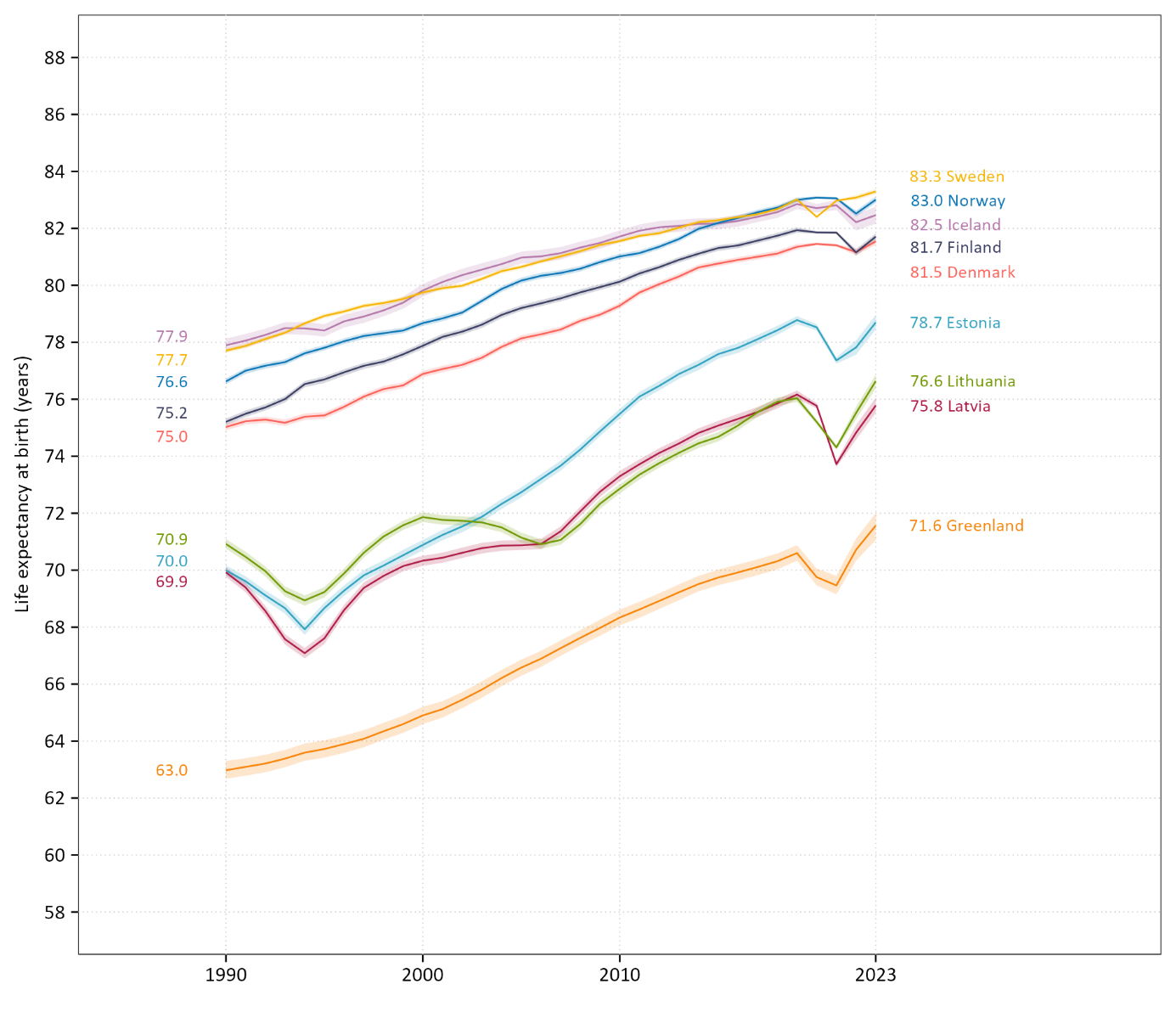


Figure S3. Life expectancy (LE) at birth in years with 95% uncertainty intervals in the Nordic and Baltic countries from 1990 to 2023. Results from GBD 2023.


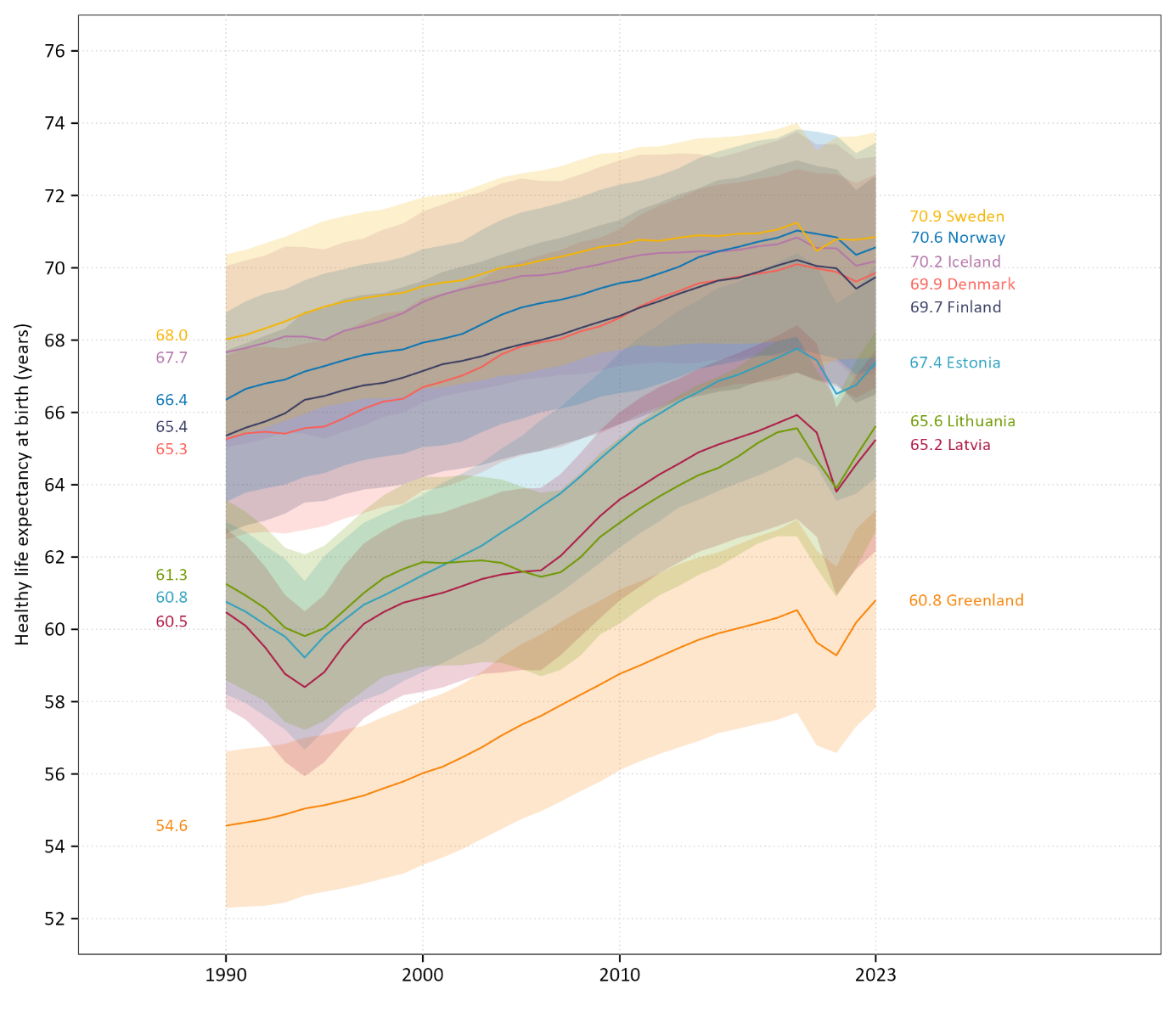


Figure S4. Heathy life expectancy (HALE) at birth in years with 95% uncertainty intervals in the Nordic and Baltic countries from 1990 to 2023. Results from GBD 2023.


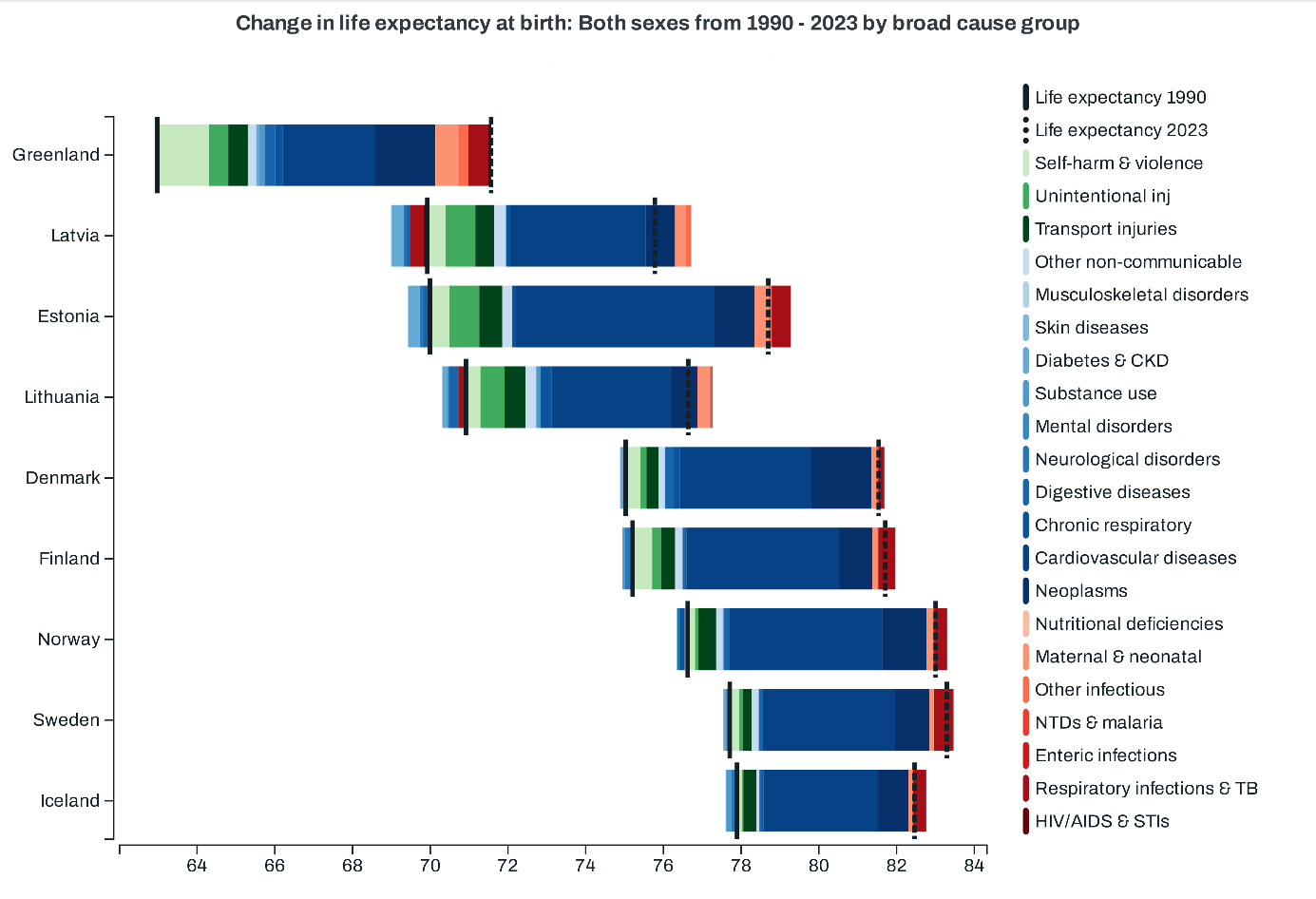


Figure S5. Change in life expectancy at birth from 1990 to 2023 by broad cause group.

Footnote Figure S5: The solid line represents life expectancy at birth in 1990, while the dashed line shows life expectancy in 2023. Contributions of specific causes to changes in life expectancy are depicted with color-coded segments. Segments between the lines represent net changes from 1990 to 2023, while segments beyond the solid (left) or dashed (right) lines indicate additional negative or positive effects, respectively, not fully reflected in these benchmarks. In the case of Latvia, for example, the interval for respiratory diseases is below the solid line for 1990 because respiratory diseases have negatively contributed to life expectancy between 1990 and 2023. Created by Institute for Health Metrics and Evaluation (IHME).

Table S3. Count, rates and age-standardised rates per 100 000 inhabitants and rank order (based on counts) for deaths and disability-adjusted life-years (DALYs) attributed to dietary risks in the Nordic and Baltic countries by country and for all countries, 2023. 95% uncertainty intervals shown in parentheses.

|  | **Deaths** | | | **DALYs** | | | **Rank** | |
| --- | --- | --- | --- | --- | --- | --- | --- | --- |
| **Dietary risk** | **Count** | **Crude rate** | **Age-standardised rate** | **Count** | **Crude rate** | **Age-standardised rate** | **Deaths** | **DALYs** |
| **Denmark (N=5 909 103·6)** |  |  |  |  |  |  |  |  |
| Dietary risks | 5233.8 (1308.7 to 8035.7) | 88.6 (22.1 to 136.0) | 38.8 (9.2 to 59.0) | 103070.9 (27332.5 to 151759.8) | 1744.3 (462.5 to 2568.2) | 882.0 (243.6 to 1279.4) | n.a. | n.a. |
| Diet low in whole grains | 1053.3 (455.4 to 1648.1) | 17.8 (7.7 to 27.9) | 7.9 (3.5 to 12.2) | 20438.7 (8555.4 to 31853.0) | 345.9 (144.8 to 539.0) | 176.3 (75.1 to 273.2) | 1 | 1 |
| Diet high in processed meat | 760.1 (407.3 to 1347.6) | 12.9 (6.9 to 22.8) | 5.8 (2.9 to 10.4) | 18338.2 (10082.6 to 31311.8) | 310.3 (170.6 to 529.9) | 165.2 (87.5 to 283.0) | 2 | 2 |
| Diet high in red meat | 809.8 (0.0 to 1597.2) | 13.7 (0.0 to 27.0) | 6.1 (0.0 to 11.8) | 17050.6 (0.0 to 33127.0) | 288.5 (0.0 to 560.6) | 149.2 (0.0 to 280.9) | 3 | 3 |
| Diet low in fruits | 818.9 (351.1 to 1381.4) | 13.9 (5.9 to 23.4) | 6.1 (2.5 to 10.3) | 16011.0 (6160.0 to 27228.3) | 271.0 (104.2 to 460.8) | 139.7 (52.4 to 237.6) | 4 | 4 |
| Diet high in sodium | 540.3 (0.5 to 1994.0) | 9.1 (0.0 to 33.7) | 4.0 (0.0 to 14.4) | 10157.4 (19.1 to 36342.2) | 171.8 (0.3 to 615.0) | 84.5 (0.2 to 291.3) | 5 | 5 |
| Diet low in vegetables | 570.0 (271.9 to 920.7) | 9.6 (4.6 to 15.6) | 4.2 (1.9 to 6.8) | 10149.5 (4349.2 to 16730.6) | 171.8 (73.6 to 283.1) | 84.3 (34.3 to 140.0) | 6 | 6 |
| Diet low in legumes | 514.9 (-447.1 to 1372.3) | 8.7 (-7.6 to 23.2) | 4.0 (-3.5 to 10.4) | 10006.5 (-8746.1 to 26013.9) | 169.3 (-148.0 to 440.2) | 89.6 (-80.1 to 227.5) | 7 | 7 |
| Diet low in nuts and seeds | 459.6 (114.8 to 864.1) | 7.8 (1.9 to 14.6) | 3.5 (0.9 to 6.6) | 8614.4 (2117.8 to 15758.4) | 145.8 (35.8 to 266.7) | 76.6 (18.9 to 140.8) | 8 | 8 |
| Diet low in omega-6 polyunsaturated fatty acids | 391.8 (-994.3 to 1595.6) | 6.6 (-16.8 to 27.0) | 3.0 (-7.7 to 12.0) | 7572.7 (-19891.9 to 29614.7) | 128.2 (-336.6 to 501.2) | 67.5 (-185.3 to 259.6) | 9 | 9 |
| Diet high in sugar-sweetened beverages | 190.0 (101.4 to 285.9) | 3.2 (1.7 to 4.8) | 1.5 (0.8 to 2.2) | 5524.1 (2884.4 to 8634.6) | 93.5 (48.8 to 146.1) | 52.8 (27.5 to 86.8) | 12 | 10 |
| Diet low in fibre | 241.3 (53.3 to 553.2) | 4.1 (0.9 to 9.4) | 1.8 (0.4 to 4.1) | 4390.9 (985.4 to 10159.5) | 74.3 (16.7 to 171.9) | 38.8 (9.5 to 89.3) | 11 | 11 |
| Diet low in milk | 245.2 (58.5 to 482.6) | 4.1 (1.0 to 8.2) | 1.8 (0.4 to 3.6) | 4277.7 (1021.3 to 8500.4) | 72.4 (17.3 to 143.9) | 35.2 (8.5 to 69.2) | 10 | 12 |
| Diet low in seafood omega-3 fatty acids | 184.8 (31.2 to 442.4) | 3.1 (0.5 to 7.5) | 1.4 (0.2 to 3.3) | 3270.5 (518.9 to 8139.2) | 55.3 (8.8 to 137.7) | 28.5 (4.4 to 71.2) | 13 | 13 |
| Diet low in calcium | 81.8 (54.9 to 113.1) | 1.4 (0.9 to 1.9) | 0.6 (0.4 to 0.8) | 1356.9 (921.0 to 1858.1) | 23.0 (15.6 to 31.4) | 10.9 (7.4 to 14.9) | 14 | 14 |
| Diet high in trans fatty acids | 0.0 (0.0 to 0.0) | 0.0 (0.0 to 0.0) | 0.0 (0.0 to 0.0) | 0.0 (0.0 to 0.0) | 0.0 (0.0 to 0.0) | 0.0 (0.0 to 0.0) | 15 | 15 |
| **Estonia (N=1 353 266·6)** |  |  |  |  |  |  |  |  |
| Dietary risks | 2967.8 (1463.3 to 4246.1) | 219.3 (108.1 to 313.8) | 91.3 (43.2 to 129.7) | 51487.4 (23498.4 to 71532.8) | 3804.7 (1736.4 to 5285.9) | 1827.9 (794.2 to 2553.8) | n.a. | n.a. |
| Diet low in fruits | 910.0 (617.8 to 1275.5) | 67.2 (45.7 to 94.3) | 27.5 (18.3 to 38.5) | 14319.8 (9390.2 to 20404.2) | 1058.1 (693.9 to 1507.8) | 498.5 (317.8 to 717.9) | 1 | 1 |
| Diet low in vegetables | 794.0 (553.1 to 1103.2) | 58.7 (40.9 to 81.5) | 23.5 (16.3 to 32.6) | 11449.9 (8050.8 to 15689.0) | 846.1 (594.9 to 1159.3) | 394.1 (163.7 to 616.2) | 2 | 2 |
| Diet high in processed meat | 419.7 (148.2 to 735.3) | 31.0 (11.0 to 54.3) | 13.8 (4.8 to 23.9) | 10249.7 (4353.3 to 16276.5) | 757.3 (321.7 to 1202.8) | 380.8 (267.1 to 518.7) | 3 | 3 |
| Diet low in whole grains | 308.5 (136.9 to 509.9) | 22.8 (10.1 to 37.7) | 9.9 (4.4 to 16.4) | 5982.8 (2497.4 to 9943.0) | 442.1 (184.5 to 734.7) | 223.5 (93.0 to 371.6) | 4 | 4 |
| Diet low in nuts and seeds | 283.6 (75.5 to 491.4) | 21.0 (5.6 to 36.3) | 9.0 (2.4 to 15.4) | 4997.8 (1328.5 to 8555.3) | 369.3 (98.2 to 632.2) | 184.3 (48.4 to 312.6) | 5 | 5 |
| Diet low in legumes | 226.7 (-182.4 to 611.9) | 16.8 (-13.5 to 45.2) | 7.2 (-5.9 to 19.1) | 4038.7 (-3359.6 to 10602.0) | 298.4 (-248.3 to 783.5) | 149.2 (-130.9 to 385.4) | 6 | 6 |
| Diet high in red meat | 156.5 (0.0 to 322.2) | 11.6 (0.0 to 23.8) | 5.3 (0.0 to 10.7) | 3692.3 (0.0 to 7551.2) | 272.8 (0.0 to 550.6) | 144.6 (0.0 to 287.2) | 9 | 7 |
| Diet low in omega-6 polyunsaturated fatty acids | 206.0 (-572.8 to 826.1) | 15.2 (-42.3 to 61.0) | 6.6 (-18.4 to 26.0) | 3678.4 (-10470.3 to 14334.0) | 271.8 (-773.7 to 1059.2) | 135.8 (-397.9 to 520.2) | 7 | 8 |
| Diet high in sodium | 188.6 (0.0 to 964.8) | 13.9 (0.0 to 71.3) | 5.9 (0.0 to 29.5) | 3187.3 (0.0 to 15598.8) | 235.5 (0.0 to 1152.7) | 112.6 (0.0 to 537.3) | 8 | 9 |
| Diet low in seafood omega-3 fatty acids | 115.7 (27.9 to 240.0) | 8.6 (2.1 to 17.7) | 3.2 (0.8 to 6.6) | 1404.1 (340.1 to 3066.9) | 103.8 (25.1 to 226.6) | 40.2 (9.6 to 90.3) | 10 | 10 |
| Diet high in sugar-sweetened beverages | 26.6 (7.0 to 72.2) | 2.0 (0.5 to 5.3) | 0.9 (0.2 to 2.5) | 881.0 (240.6 to 2150.1) | 65.1 (17.8 to 158.9) | 36.6 (9.8 to 89.2) | 13 | 11 |
| Diet low in fibre | 49.6 (6.8 to 137.9) | 3.7 (0.5 to 10.2) | 1.5 (0.2 to 4.2) | 855.3 (108.1 to 2537.0) | 63.2 (8.0 to 187.5) | 31.2 (3.8 to 92.1) | 11 | 12 |
| Diet low in milk | 41.2 (9.0 to 85.4) | 3.0 (0.7 to 6.3) | 1.3 (0.3 to 2.8) | 747.1 (160.3 to 1568.4) | 55.2 (11.8 to 115.9) | 27.2 (5.8 to 57.0) | 12 | 13 |
| Diet low in calcium | 15.4 (10.7 to 20.4) | 1.1 (0.8 to 1.5) | 0.5 (0.3 to 0.6) | 265.5 (183.7 to 351.1) | 19.6 (13.6 to 25.9) | 9.4 (6.5 to 12.5) | 14 | 14 |
| Diet high in trans fatty acids | 0.0 (0.0 to 0.0) | 0.0 (0.0 to 0.0) | 0.0 (0.0 to 0.0) | 0.0 (0.0 to 0.0) | 0.0 (0.0 to 0.0) | 0.0 (0.0 to 0.0) | 15 | 15 |
| **Finland (N=5 552 897·5)** |  |  |  |  |  |  |  |  |
| Dietary risks | 8220.8 (1092.7 to 13096.9) | 148.0 (19.7 to 235.9) | 54.6 (7.6 to 85.3) | 148458.6 (25690.6 to 224907.1) | 2673.5 (462.7 to 4050.3) | 1202.0 (235.9 to 1769.0) | n.a. | n.a. |
| Diet low in fruits | 1622.3 (881.8 to 2437.0) | 29.2 (15.9 to 43.9) | 10.7 (5.6 to 16.2) | 27297.4 (13052.3 to 42545.2) | 491.6 (235.1 to 766.2) | 222.0 (99.0 to 347.9) | 1 | 1 |
| Diet low in whole grains | 1290.1 (579.9 to 2005.8) | 23.2 (10.4 to 36.1) | 8.8 (4.0 to 13.6) | 23831.3 (9966.8 to 37603.0) | 429.2 (179.5 to 677.2) | 197.9 (82.0 to 309.0) | 3 | 2 |
| Diet low in vegetables | 1404.7 (967.0 to 1921.4) | 25.3 (17.4 to 34.6) | 9.0 (6.0 to 12.3) | 21080.8 (13539.2 to 29243.2) | 379.6 (243.8 to 526.6) | 160.0 (97.2 to 223.7) | 2 | 3 |
| Diet high in red meat | 871.7 (0.0 to 1602.0) | 15.7 (0.0 to 28.9) | 6.2 (0.0 to 11.0) | 20664.2 (0.0 to 37048.4) | 372.1 (0.0 to 667.2) | 181.1 (0.0 to 316.0) | 8 | 4 |
| Diet low in nuts and seeds | 1235.9 (313.2 to 2186.6) | 22.3 (5.6 to 39.4) | 8.3 (2.1 to 14.5) | 20324.4 (5040.6 to 35432.6) | 366.0 (90.8 to 638.1) | 164.3 (41.1 to 287.1) | 4 | 5 |
| Diet low in legumes | 1089.3 (-926.4 to 2953.1) | 19.6 (-16.7 to 53.2) | 7.4 (-6.3 to 19.7) | 18279.2 (-15332.1 to 48510.2) | 329.2 (-276.1 to 873.6) | 148.8 (-126.0 to 383.4) | 5 | 6 |
| Diet high in sodium | 986.8 (2.1 to 3480.6) | 17.8 (0.0 to 62.7) | 6.6 (0.0 to 22.9) | 16838.8 (102.2 to 56925.7) | 303.2 (1.8 to 1025.2) | 133.8 (1.6 to 437.1) | 6 | 7 |
| Diet high in processed meat | 562.5 (187.9 to 1037.3) | 10.1 (3.4 to 18.7) | 4.1 (1.3 to 7.7) | 16671.0 (8407.4 to 29472.5) | 300.2 (151.4 to 530.8) | 155.5 (74.1 to 280.5) | 9 | 8 |
| Diet low in omega-6 polyunsaturated fatty acids | 890.1 (-2490.9 to 3576.7) | 16.0 (-44.9 to 64.4) | 6.0 (-17.0 to 23.8) | 14886.4 (-41837.5 to 58480.9) | 268.1 (-753.4 to 1053.2) | 120.4 (-348.9 to 463.6) | 7 | 9 |
| Diet high in sugar-sweetened beverages | 187.1 (70.7 to 358.8) | 3.4 (1.3 to 6.5) | 1.4 (0.5 to 2.6) | 6790.6 (3167.5 to 13293.8) | 122.3 (57.0 to 239.4) | 67.2 (30.5 to 131.4) | 11 | 10 |
| Diet low in fibre | 392.4 (124.0 to 826.6) | 7.1 (2.2 to 14.9) | 2.5 (0.8 to 5.4) | 6143.0 (1737.1 to 13701.7) | 110.6 (31.3 to 246.7) | 48.6 (12.6 to 109.5) | 10 | 11 |
| Diet low in milk | 87.1 (17.9 to 182.3) | 1.6 (0.3 to 3.3) | 0.6 (0.1 to 1.3) | 1525.5 (311.3 to 3208.9) | 27.5 (5.6 to 57.8) | 12.3 (2.5 to 25.9) | 13 | 12 |
| Diet low in seafood omega-3 fatty acids | 109.5 (3.5 to 410.2) | 2.0 (0.1 to 7.4) | 0.7 (0.0 to 2.5) | 1430.9 (41.9 to 5680.4) | 25.8 (0.8 to 102.3) | 9.8 (0.2 to 40.2) | 12 | 13 |
| Diet low in calcium | 51.5 (34.7 to 71.3) | 0.9 (0.6 to 1.3) | 0.4 (0.2 to 0.5) | 879.7 (605.6 to 1205.5) | 15.8 (10.9 to 21.7) | 7.0 (4.9 to 9.5) | 14 | 14 |
| Diet high in trans fatty acids | 0.0 (0.0 to 0.0) | 0.0 (0.0 to 0.0) | 0.0 (0.0 to 0.0) | 0.0 (0.0 to 0.0) | 0.0 (0.0 to 0.0) | 0.0 (0.0 to 0.0) | 15 | 15 |
| **Greenland (N=56 609·6)** |  |  |  |  |  |  |  |  |
| Dietary risks | 44.3 (22.4 to 65.0) | 78.3 (39.6 to 114.8) | 84.4 (42.5 to 126.3) | 1112.7 (582.5 to 1591.4) | 1965.5 (1029.0 to 2811.1) | 1723.5 (895.8 to 2531.3) | n.a. | n.a. |
| Diet high in processed meat | 10.8 (4.4 to 18.1) | 19.0 (7.8 to 32.0) | 19.4 (8.4 to 33.0) | 298.6 (123.2 to 477.2) | 527.5 (217.7 to 842.9) | 443.8 (190.8 to 722.8) | 1 | 1 |
| Diet low in whole grains | 9.9 (4.2 to 16.0) | 17.5 (7.4 to 28.2) | 18.7 (7.8 to 30.3) | 243.4 (99.6 to 394.4) | 430.0 (176.0 to 696.7) | 376.3 (152.0 to 606.4) | 2 | 2 |
| Diet high in red meat | 6.6 (0.0 to 11.5) | 11.7 (0.0 to 20.3) | 12.2 (0.0 to 22.5) | 173.2 (0.0 to 289.9) | 305.9 (0.0 to 512.2) | 259.7 (0.0 to 449.1) | 3 | 3 |
| Diet low in fruits | 5.0 (1.4 to 10.9) | 8.8 (2.4 to 19.2) | 9.2 (2.5 to 20.3) | 128.1 (38.3 to 278.4) | 226.4 (67.7 to 491.7) | 197.6 (59.9 to 415.0) | 6 | 4 |
| Diet high in sodium | 5.3 (0.0 to 20.2) | 9.4 (0.0 to 35.6) | 10.2 (0.0 to 40.2) | 124.2 (0.2 to 464.4) | 219.4 (0.3 to 820.4) | 196.6 (0.2 to 742.1) | 4 | 5 |
| Diet low in vegetables | 5.1 (1.0 to 9.2) | 9.0 (1.8 to 16.2) | 9.9 (2.6 to 17.8) | 119.1 (22.0 to 216.1) | 210.4 (38.9 to 381.7) | 188.9 (41.7 to 335.8) | 5 | 6 |
| Diet high in sugar-sweetened beverages | 2.6 (0.8 to 5.5) | 4.5 (1.4 to 9.7) | 4.6 (1.4 to 9.5) | 87.3 (27.7 to 168.7) | 154.2 (48.9 to 298.0) | 127.9 (41.1 to 252.4) | 9 | 7 |
| Diet low in legumes | 3.4 (-2.6 to 9.4) | 5.9 (-4.5 to 16.5) | 6.5 (-4.8 to 18.1) | 80.6 (-63.5 to 215.0) | 142.4 (-112.2 to 379.8) | 126.6 (-98.5 to 347.4) | 7 | 8 |
| Diet low in milk | 3.1 (0.8 to 6.1) | 5.5 (1.4 to 10.7) | 5.9 (1.6 to 11.5) | 67.7 (17.7 to 132.4) | 119.6 (31.2 to 234.0) | 107.2 (28.3 to 209.0) | 8 | 9 |
| Diet low in omega-6 polyunsaturated fatty acids | 1.5 (-3.1 to 7.8) | 2.7 (-5.5 to 13.8) | 3.0 (-6.3 to 15.4) | 37.1 (-76.4 to 183.4) | 65.6 (-134.9 to 324.0) | 58.2 (-122.9 to 297.8) | 10 | 10 |
| Diet low in fibre | 1.3 (0.0 to 5.0) | 2.4 (0.1 to 7.7) | 2.8 (0.1 to 9.8) | 32.6 (0.9 to 127.4) | 57.6 (1.7 to 225.1) | 54.4 (1.9 to 202.8) | 12 | 11 |
| Diet low in nuts and seeds | 1.4 (0.1 to 4.4) | 2.4 (0.1 to 8.9) | 2.9 (0.2 to 9.0) | 31.1 (1.6 to 99.5) | 54.9 (2.8 to 175.8) | 52.6 (3.3 to 166.9) | 11 | 12 |
| Diet low in seafood omega-3 fatty acids | 1.1 (0.1 to 3.9) | 2.0 (0.1 to 6.8) | 2.3 (0.1 to 7.7) | 26.5 (1.0 to 92.2) | 46.8 (1.8 to 162.9) | 43.6 (2.0 to 148.0) | 13 | 13 |
| Diet low in calcium | 0.8 (0.5 to 1.3) | 1.4 (0.9 to 2.3) | 1.7 (1.1 to 2.7) | 16.9 (10.5 to 26.1) | 29.8 (18.6 to 46.2) | 28.6 (17.6 to 45.2) | 14 | 14 |
| Diet high in trans fatty acids | 0.0 (0.0 to 0.0) | 0.0 (0.0 to 0.0) | 0.0 (0.0 to 0.0) | 0.0 (0.0 to 0.0) | 0.0 (0.0 to 0.0) | 0.0 (0.0 to 0.0) | 15 | 15 |
| **Iceland (N=386 545·2)** |  |  |  |  |  |  |  |  |
| Dietary risks | 281.1 (76.5 to 445.6) | 72.7 (19.8 to 115.3) | 43.3 (12.1 to 67.9) | 5845.5 (1921.3 to 8634.3) | 1512.2 (497.9 to 2233.7) | 991.8 (338.3 to 1455.9) | n.a. | n.a. |
| Diet low in whole grains | 68.1 (34.4 to 100.2) | 17.6 (8.9 to 25.9) | 10.6 (5.4 to 15.5) | 1423.9 (680.8 to 2131.7) | 364.4 (176.1 to 551.5) | 244.1 (117.6 to 364.2) | 1 | 1 |
| Diet high in processed meat | 37.1 (9.1 to 80.0) | 9.6 (2.4 to 20.7) | 5.9 (1.3 to 12.7) | 1066.5 (378.0 to 1979.3) | 275.9 (97.8 to 512.1) | 188.8 (65.1 to 347.4) | 4 | 2 |
| Diet high in red meat | 41.7 (0.0 to 77.0) | 10.8 (0.0 to 19.9) | 6.6 (0.0 to 12.0) | 1053.3 (0.0 to 1831.9) | 272.5 (0.0 to 473.9) | 183.9 (0.0 to 318.7) | 2 | 3 |
| Diet low in nuts and seeds | 38.4 (9.5 to 71.2) | 9.9 (2.5 to 18.4) | 5.9 (1.5 to 11.0) | 691.1 (171.9 to 1262.5) | 178.8 (44.5 to 326.6) | 118.0 (29.6 to 214.7) | 3 | 4 |
| Diet low in fruits | 31.1 (12.3 to 56.4) | 8.0 (3.2 to 14.6) | 4.8 (1.9 to 8.7) | 659.4 (248.5 to 1178.5) | 170.6 (64.3 to 304.9) | 114.5 (42.0 to 202.7) | 8 | 5 |
| Diet low in vegetables | 34.1 (16.3 to 51.0) | 8.8 (4.2 to 13.2) | 5.2 (2.4 to 7.8) | 651.4 (282.2 to 991.3) | 168.5 (73.0 to 256.5) | 109.4 (46.7 to 168.0) | 6 | 6 |
| Diet high in sodium | 36.4 (0.1 to 118.8) | 9.4 (0.0 to 30.7) | 5.5 (0.0 to 17.9) | 650.8 (2.8 to 2084.6) | 168.4 (0.7 to 539.3) | 105.9 (0.5 to 338.3) | 5 | 7 |
| Diet low in legumes | 34.0 (-25.4 to 90.2) | 8.8 (-6.6 to 23.3) | 5.3 (-4.0 to 13.9) | 623.2 (-493.2 to 1601.5) | 161.2 (-127.6 to 414.3) | 106.4 (-85.7 to 270.7) | 7 | 8 |
| Diet low in omega-6 polyunsaturated fatty acids | 25.6 (-58.5 to 104.0) | 6.6 (-15.1 to 26.9) | 4.0 (-9.1 to 16.0) | 473.5 (-1089.5 to 1847.4) | 122.5 (-281.9 to 477.9) | 81.0 (-188.6 to 312.5) | 9 | 9 |
| Diet low in fibre | 19.9 (5.2 to 42.4) | 5.1 (1.3 to 11.0) | 3.0 (0.8 to 6.5) | 357.8 (92.7 to 790.2) | 92.6 (24.0 to 204.4) | 61.4 (15.9 to 136.0) | 10 | 10 |
| Diet high in sugar-sweetened beverages | 6.6 (1.6 to 17.0) | 1.7 (0.4 to 4.4) | 1.1 (0.3 to 2.7) | 246.3 (60.9 to 585.8) | 63.7 (15.8 to 151.6) | 45.4 (11.4 to 107.3) | 11 | 11 |
| Diet low in milk | 4.4 (1.0 to 9.2) | 1.1 (0.3 to 2.4) | 0.7 (0.2 to 1.4) | 86.2 (19.1 to 178.7) | 22.3 (4.9 to 46.2) | 14.5 (3.2 to 29.9) | 12 | 12 |
| Diet low in calcium | 2.0 (1.4 to 2.7) | 0.5 (0.4 to 0.7) | 0.3 (0.2 to 0.4) | 38.1 (27.3 to 51.5) | 9.9 (7.1 to 13.3) | 6.4 (4.5 to 8.6) | 13 | 13 |
| Diet low in seafood omega-3 fatty acids | 0.7 (0.0 to 4.3) | 0.2 (0.0 to 1.1) | 0.1 (0.0 to 0.6) | 9.2 (0.0 to 63.0) | 2.4 (0.0 to 16.3) | 1.5 (0.0 to 10.2) | 14 | 14 |
| Diet high in trans fatty acids | 0.0 (0.0 to 0.0) | 0.0 (0.0 to 0.0) | 0.0 (0.0 to 0.0) | 0.0 (0.0 to 0.0) | 0.0 (0.0 to 0.0) | 0.0 (0.0 to 0.0) | 15 | 15 |
| **Latvia (N=1 894 106·0)** |  |  |  |  |  |  |  |  |
| Dietary risks | 4910.9 (1705.0 to 7359.3) | 259.2 (90.2 to 388.5) | 111.4 (40.2 to 164.4) | 96395.0 (36618.7 to 140543.4) | 5089.2 (1933.3 to 7520.0) | 2514.8 (1000.4 to 3581.9) | n.a. | n.a. |
| Diet low in fruits | 1151.2 (548.9 to 1812.6) | 60.8 (29.0 to 95.7) | 26.6 (12.3 to 41.5) | 22918.5 (9900.0 to 35656.8) | 1210.0 (517.4 to 1882.5) | 616.3 (248.3 to 947.4) | 1 | 1 |
| Diet high in processed meat | 955.0 (247.2 to 1692.8) | 50.4 (13.1 to 89.2) | 22.4 (5.4 to 39.2) | 22620.8 (6888.0 to 37220.6) | 1194.3 (363.7 to 1965.1) | 613.9 (174.9 to 994.9) | 2 | 2 |
| Diet low in whole grains | 702.7 (287.0 to 1212.8) | 37.1 (15.2 to 64.0) | 16.3 (6.7 to 27.6) | 14064.9 (5683.0 to 23806.8) | 742.6 (300.0 to 1256.9) | 377.1 (155.7 to 620.6) | 4 | 3 |
| Diet low in nuts and seeds | 711.9 (199.3 to 1232.8) | 37.6 (10.5 to 65.0) | 16.3 (4.7 to 27.8) | 13148.3 (3802.9 to 22278.7) | 694.2 (200.8 to 1176.2) | 350.5 (103.0 to 595.1) | 3 | 4 |
| Diet low in legumes | 635.4 (-515.3 to 1710.7) | 33.5 (-27.2 to 90.3) | 14.6 (-11.9 to 38.7) | 11937.2 (-9791.5 to 30900.5) | 630.2 (-516.9 to 1631.4) | 319.1 (-276.0 to 806.0) | 5 | 5 |
| Diet high in sodium | 585.9 (2.1 to 2121.8) | 30.9 (0.1 to 112.0) | 13.3 (0.1 to 46.8) | 11086.4 (48.8 to 37751.3) | 585.3 (2.6 to 1993.1) | 282.4 (1.3 to 934.2) | 7 | 6 |
| Diet low in vegetables | 615.3 (393.1 to 881.5) | 32.5 (20.8 to 46.5) | 13.6 (8.5 to 19.6) | 10799.1 (6468.6 to 15735.9) | 570.1 (341.5 to 830.8) | 274.1 (162.0 to 402.2) | 6 | 7 |
| Diet high in red meat | 287.3 (0.0 to 590.7) | 15.2 (0.0 to 31.2) | 7.1 (0.0 to 14.2) | 7378.8 (0.0 to 14791.7) | 389.6 (0.0 to 780.9) | 208.2 (0.0 to 400.1) | 9 | 8 |
| Diet low in omega-6 polyunsaturated fatty acids | 364.6 (-746.2 to 1537.2) | 19.2 (-39.4 to 81.2) | 8.4 (-17.3 to 35.0) | 6865.6 (-14023.4 to 27876.0) | 362.5 (-740.4 to 1471.7) | 183.9 (-380.3 to 735.2) | 8 | 9 |
| Diet low in seafood omega-3 fatty acids | 215.9 (28.3 to 523.3) | 11.4 (1.5 to 27.7) | 4.8 (0.6 to 11.8) | 3639.8 (445.4 to 9195.1) | 192.2 (23.5 to 485.5) | 93.3 (11.1 to 240.8) | 10 | 10 |
| Diet low in fibre | 179.3 (29.1 to 470.2) | 9.5 (1.5 to 24.8) | 3.9 (0.6 to 10.4) | 3066.1 (481.9 to 8357.2) | 161.9 (25.4 to 441.2) | 79.9 (12.3 to 218.9) | 11 | 11 |
| Diet low in milk | 95.9 (25.01to 171.1) | 5.1 (1.3 to 9.0) | 2.2 (0.6 to 3.9) | 1889.2 (494.0 to 3371.4) | 99.7 (26.1 to 178.0) | 49.1 (12.9 to 87.3) | 12 | 12 |
| Diet high in sugar-sweetened beverages | 38.9 (10.2 to 110.2) | 2.1 (0.5 to 5.8) | 1.0 (0.2 to 2.7) | 1210.7 (305.5 to 3116.3) | 63.9 (16.1 to 165.5) | 35.8 (8.9 to 90.9) | 13 | 13 |
| Diet low in calcium | 27.1 (18.6 to 37.5) | 1.4 (1.0 to 2.0) | 0.6 (0.4 to 0.8) | 484.5 (331.3 to 676.1) | 25.6 (17.5 to 35.7) | 12.0 (8.2 to 16.7) | 14 | 14 |
| Diet high in trans fatty acids | 0.0 (0.0 to 0.0) | 0.0 (0.0 to 0.0) | 0.0 (0.0 to 0.0) | 0.0 (0.0 to 0.0) | 0.0 (0.0 to 0.0) | 0.0 (0.0 to 0.0) | 15 | 15 |
| **Lithuania (N=2 839 078·2)** |  |  |  |  |  |  |  |  |
| Dietary risks | 7664.8 (1742.6 to 12002.8) | 270.0 (61.4 to 422.8) | 118.5 (28.6 to 183.4) | 145365.7 (40481.7 to 215064.2) | 5120.2 (1425.9 to 7575.1) | 2503.3 (743.3 to 3653.0) | n.a. | n.a. |
| Diet high in processed meat | 1473.8 (159.6 to 2788.7) | 51.9 (5.6 to 98.2) | 23.3 (2.5 to 43.7) | 33142.0 (6389.5 to 57696.8) | 1167.3 (225.1 to 2032.2) | 590.1 (113.3 to 1005.7) | 2 | 1 |
| Diet low in fruits | 1535.3 (566.3 to 2549.5) | 54.1 (19.9 to 89.8) | 24.3 (9.0 to 40.1) | 30644.9 (11151.0 to 49638.4) | 1079.4 (392.8 to 1748.4) | 550.5 (197.1 to 881.2) | 1 | 2 |
| Diet low in whole grains | 1204.4 (573.9 to 1896.4) | 42.4 (20.2 to 66.8) | 18.8 (8.9 to 29.9) | 22833.0 (10217.2 to 37230.6) | 806.0 (359.9 to 1311.4) | 401.0 (179.0 to 652.7) | 4 | 3 |
| Diet low in nuts and seeds | 1257.3 (337.8 to 2182.3) | 44.3 (11.9 to 76.9) | 19.3 (5.3 to 33.3) | 21724.0 (6071.8 to 37076.1) | 765.2 (213.9 to 1305.9) | 373.1 (106.4 to 641.7) | 3 | 4 |
| Diet low in legumes | 1094.6 (-890.9 to 2869.8) | 38.6 (-31.4 to 101.1) | 16.9 (-13.9 to 44.0) | 19252.8 (-15994.0 to 49802.3) | 678.1 (-563.4 to 1754.2) | 331.1 (-281.1 to 848.9) | 5 | 5 |
| Diet high in red meat | 630.2 (0.0 to 1364.2) | 22.2 (0.0 to 48.1) | 10.3 (0.0 to 21.6) | 15186.0 (0.0 to 31867.2) | 534.9 (0.0 to 1087.2) | 276.9 (0.0 to 543.6) | 9 | 6 |
| Diet low in omega-6 polyunsaturated fatty acids | 828.7 (-1986.6 to 3262.7) | 29.2 (-70.0 to 115.0) | 12.8 (-31.0 to 49.9) | 14630.9 (-35931.4 to 55974.2) | 515.3 (-1265.6 to 1971.6) | 251.7 (-625.4 to 956.2) | 6 | 7 |
| Diet low in vegetables | 765.4 (460.4 to 1137.7) | 27.0 (16.2 to 40.1) | 11.9 (7.1 to 18.0) | 14086.2 (8120.1 to 21308.9) | 496.2 (286.0 to 750.6) | 245.9 (140.3 to 372.2) | 8 | 8 |
| Diet high in sodium | 803.0 (0.0 to 2962.7) | 28.3 (0.0 to 104.4) | 12.2 (0.0 to 44.9) | 14053.7 (1.0 to 52099.9) | 495.0 (0.0 to 1835.1) | 233.7 (0.0 to 867.2) | 7 | 9 |
| Diet low in fibre | 344.3 (58.3 to 866.9) | 12.1 (2.1 to 30.5) | 5.2 (0.9 to 13.2) | 5576.5 (885.2 to 14609.6) | 196.4 (31.2 to 514.6) | 95.7 (15.4 to 258.1) | 10 | 10 |
| Diet low in seafood omega-3 fatty acids | 257.7 (27.8 to 720.2) | 9.1 (1.0 to 25.4) | 3.8 (0.4 to 10.8) | 3967.4 (386.0 to 11147.8) | 139.7 (13.6 to 392.7) | 65.8 (6.2 to 189.0) | 11 | 11 |
| Diet high in sugar-sweetened beverages | 90.6 (19.3 to 260.9) | 3.2 (0.7 to 9.2) | 1.5 (0.3 to 4.3) | 2711.3 (658.7 to 6546.4) | 95.5 (23.2 to 230.6) | 52.3 (12.6 to 125.4) | 13 | 12 |
| Diet low in milk | 102.8 (23.8 to 201.0) | 3.6 (0.8 to 7.1) | 1.6 (0.4 to 3.2) | 2019.7 (469.4 to 3980.4) | 71.1 (16.5 to 140.2) | 35.7 (8.3 to 70.4) | 12 | 13 |
| Diet low in calcium | 38.6 (25.4 to 52.2) | 1.4 (0.9 to 1.8) | 0.6 (0.4 to 0.8) | 690.8 (461.9 to 931.2) | 24.3 (16.3 to 32.8) | 11.7 (7.9 to 15.8) | 14 | 14 |
| Diet high in trans fatty acids | 0.0 (0.0 to 0.0) | 0.0 (0.0 to 0.0) | 0.0 (0.0 to 0.0) | 0.0 (0.0 to 0.0) | 0.0 (0.0 to 0.0) | 0.0 (0.0 to 0.0) | 15 | 15 |
| **Norway (N=5 470 981·5)** |  |  |  |  |  |  |  |  |
| Dietary risks | 3890.2 (1747.5 to 5864.6) | 71.1 (31.9 to 105.4) | 33.7 (15.5 to 49.3) | 83249.1 (41180.0 to 118006.3) | 1521.6 (752.7 to 2157.0) | 837.4 (433.9 to 1178.7) | n.a. | n.a. |
| Diet high in processed meat | 927.3 (444.5 to 1544.2) | 17.0 (8.1 to 28.2) | 8.2 (3.8 to 13.5) | 24197.8 (12601.8 to 35607.0) | 442.3 (230.3 to 650.8) | 252.2 (132.3 to 370.2) | 1 | 1 |
| Diet low in whole grains | 910.7 (432.8 to 1409.8) | 16.6 (7.9 to 25.8) | 7.9 (3.8 to 12.2) | 18345.7 (8031.2 to 29039.4) | 353.3 (146.8 to 530.8) | 183.3 (80.1 to 289.1) | 2 | 2 |
| Diet high in red meat | 607.7 (0.0 to 1129.4) | 11.1 (0.0 to 20.6) | 5.4 (0.0 to 9.9) | 14868.8 (0.0 to 26161.3) | 271.8 (0.0 to 478.2) | 154.8 (0.0 to 272.3) | 3 | 3 |
| Diet low in fruits | 418.5 (170.5 to 743.1) | 7.6 (3.1 to 13.6) | 3.7 (1.5 to 6.5) | 8522.0 (3076.3 to 15549.5) | 155.8 (56.2 to 284.2) | 87.9 (31.8 to 161.1) | 5 | 4 |
| Diet low in vegetables | 459.1 (240.7 to 695.1) | 8.4 (4.4 to 12.7) | 3.9 (1.9 to 6.0) | 8227.4 (3856.8 to 12755.5) | 150.4 (70.5 to 233.1) | 79.7 (35.4 to 124.1) | 4 | 5 |
| Diet low in nuts and seeds | 378.3 (89.6 to 715.9) | 6.9 (1.6 to 13.1) | 3.3 (0.8 to 6.2) | 7001.3 (1674.8 to 13649.6) | 128.0 (30.6 to 249.5) | 70.1 (17.0 to 136.5) | 6 | 6 |
| Diet high in sodium | 319.3 (0.7 to 1258.2) | 5.8 (0.0 to 23.0) | 2.7 (0.0 to 10.6) | 6021.6 (18.5 to 22526.7) | 110.1 (0.3 to 411.7) | 57.9 (0.2 to 213.0) | 7 | 7 |
| Diet low in legumes | 293.5 (-218.4 to 798.2) | 5.4 (-4.0 to 14.6) | 2.6 (-1.9 to 6.9) | 5557.1 (-4347.9 to 14634.3) | 101.6 (-77.6 to 267.5) | 55.8 (-43.7 to 144.8) | 8 | 8 |
| Diet high in sugar-sweetened beverages | 123.9 (54.2 to 226.2) | 2.3 (1.0 to 4.1) | 1.1 (0.5 to 2.1) | 4889.3 (2163.5 to 9272.8) | 89.4 (39.5 to 169.5) | 55.6 (24.8 to 105.5) | 12 | 9 |
| Diet low in milk | 197.1 (49.7 to 380.6) | 3.6 (0.9 to 7.0) | 1.7 (0.4 to 3.4) | 3578.7 (895.3 to 6914.2) | 65.4 (16.4 to 126.4) | 35.2 (8.8 to 67.7) | 9 | 10 |
| Diet low in omega-6 polyunsaturated fatty acids | 154.3 (-255.1 to 689.8) | 2.8 (-4.7 to 12.6) | 1.3 (-2.2 to 6.1) | 2940.3 (-4764.2 to 13252.2) | 53.7 (-87.1 to 242.2) | 29.7 (-48.4 to 134.2) | 11 | 11 |
| Diet low in fibre | 163.0 (34.0 to 389.1) | 3.0 (0.6 to 7.1) | 1.4 (0.3 to 3.3) | 2857.7 (519.0 to 7310.9) | 52.2 (9.5 to 133.6) | 28.1 (4.9 to 72.8) | 10 | 12 |
| Diet low in calcium | 69.2 (47.5 to 95.8) | 1.3 (0.9 to 1.8) | 0.6 (0.4 to 0.8) | 1170.3 (813.4 to 1624.8) | 21.4 (14.9 to 29.7) | 11.2 (7.8 to 15.6) | 13 | 13 |
| Diet low in seafood omega-3 fatty acids | 31.6 (0.7 to 133.7) | 0.6 (0.0 to 2.4) | 0.3 (0.0 to 1.1) | 508.5 (8.4 to 2216.6) | 9.3 (0.2 to 40.5) | 4.9 (0.1 to 21.5) | 14 | 14 |
| Diet high in trans fatty acids | 0.0 (0.0 to 0.0) | 0.0 (0.0 to 0.0) | 0.0 (0.0 to 0.0) | 0.0 (0.0 to 0.0) | 0.0 (0.0 to 0.0) | 0.0 (0.0 to 0.0) | 15 | 15 |
| **Sweden (N=10 601 432·6)** |  |  |  |  |  |  |  |  |
| Dietary risks | 10827.9 (3001.6 to 16779.1) | 102.1 (28.3 to 158.3) | 41.7 (12.1 to 63.6) | 208883.5 (68375.0 to 305495.8) | 1970.3 (645.0 to 2881.6) | 979.8 (345.3 to 1400.5) | n.a. | n.a. |
| Diet high in processed meat | 1771.8 (782.1 to 2948.8) | 16.7 (7.4 to 27.8) | 7.2 (3.0 to 12.1) | 45844.9 (23465.3 to 69502.3) | 432.4 (221.3 to 655.6) | 233.9 (119.7 to 350.1) | 2 | 1 |
| Diet low in whole grains | 2163.9 (1046.3 to 3433.3) | 20.4 (9.9 to 32.4) | 8.5 (4.2 to 13.4) | 40321.7 (18327.3 to 65955.5) | 380.3 (172.9 to 622.1) | 191.0 (87.4 to 314.5) | 1 | 2 |
| Diet high in red meat | 1454.2 (0.0 to 2688.1) | 13.7 (0.0 to 25.4) | 6.0 (0.0 to 10.9) | 33970.0 (0.0 to 62164.0) | 320.4 (0.0 to 586.4) | 171.3 (0.0 to 314.2) | 4 | 3 |
| Diet low in fruits | 1666.7 (793.2 to 2641.3) | 15.7 (7.5 to 24.9) | 6.4 (2.9 to 10.2) | 30518.3 (12690.2 to 49993.4) | 287.9 (119.7 to 471.6) | 143.3 (57.0 to 236.2) | 3 | 4 |
| Diet low in vegetables | 1390.6 (875.3 to 1979.8) | 13.1 (8.3 to 18.7) | 5.1 (3.1 to 7.3) | 22330.9 (13240.4 to 31828.2) | 210.6 (124.9 to 300.2) | 97.0 (53.8 to 141.0) | 5 | 5 |
| Diet high in sodium | 1189.3 (4.9 to 4033.1) | 11.2 (0.0 to 38.0) | 4.4 (0.0 to 14.7) | 20295.6 (128.5 to 66154.8) | 191.4 (1.2 to 624.0) | 87.3 (0.7 to 284.5) | 6 | 6 |
| Diet low in legumes | 1129.4 (-906.4 to 3042.0) | 10.7 (-8.5 to 28.7) | 4.4 (-3.6 to 11.8) | 19326.8 (-15664.8 to 50283.2) | 182.3 (-147.8 to 474.3) | 91.3 (-75.8 to 231.5) | 7 | 7 |
| Diet low in nuts and seeds | 1113.3 (276.0 to 2039.6) | 10.5 (2.6 to 19.2) | 4.3 (1.1 to 7.9) | 18474.4 (4437.5 to 33996.2) | 174.3 (41.9 to 320.7) | 86.0 (20.9 to 156.9) | 8 | 8 |
| Diet low in omega-6 polyunsaturated fatty acids | 934.7 (-2326.8 to 3839.5) | 8.8 (-21.9 to 36.2) | 3.7 (-9.2 to 14.8) | 15871.6 (-40068.3 to 62117.8) | 149.7 (-378.0 to 585.9) | 74.5 (-186.2 to 285.4) | 9 | 9 |
| Diet high in sugar-sweetened beverages | 326.4 (147.8 to 597.3) | 3.1 (1.4 to 5.6) | 1.3 (0.6 to 2.4) | 11430.8 (5448.7 to 21226.1) | 107.8 (51.4 to 200.2) | 63.3 (31.7 to 114.8) | 11 | 10 |
| Diet low in fibre | 630.2 (245.0 to 1174.1) | 5.9 (2.3 to 11.1) | 2.3 (0.9 to 4.3) | 9881.6 (3922.0 to 18186.5) | 93.2 (37.0 to 171.5) | 44.6 (17.0 to 82.2) | 10 | 11 |
| Diet low in milk | 298.8 (70.2 to 619.8) | 2.8 (0.7 to 5.8) | 1.2 (0.3 to 2.5) | 5416.2 (1258.9 to 11098.5) | 51.1 (11.6 to 104.7) | 25.9 (6.0 to 53.9) | 12 | 12 |
| Diet low in seafood omega-3 fatty acids | 215.8 (15.0 to 651.6) | 2.8 (0.1 to 6.1) | 0.8 (0.1 to 2.4) | 3044.9 (174.9 to 9383.3) | 28.7 (1.6 to 88.5) | 12.9 (0.7 to 40.8) | 13 | 13 |
| Diet low in calcium | 113.0 (75.6 to 157.4) | 1.1 (0.7 to 1.5) | 0.4 (0.3 to 0.6) | 1922.8 (1306.5 to 2658.6) | 18.1 (12.3 to 25.1) | 8.8 (6.0 to 12.0) | 14 | 14 |
| Diet high in trans fatty acids | 0.0 (0.0 to 0.0) | 0.0 (0.0 to 0.0) | 0.0 (0.0 to 0.0) | 0.0 (0.0 to 0.0) | 0.0 (0.0 to 0.0) | 0.0 (0.0 to 0.0) | 15 | 15 |

# Contributions

## Providing data or critical feedback on data sources

Rune Blomhoff, Michael Brauer, Demewoz Haile, Rasmus J Havmoeller, Nityanand Jain, Lars Johansson, Mikk Jürisson, Adnan Kisa, Ann Kristin Skrindo Knudsen, Christian Madsen, Gavin Pereira, Tagli Pitsi, Tommi Juhani Vasankari, and Eva Warensjö Lemming.

## Developing methods or computational machinery

Ann Kristin Skrindo Knudsen.

## Providing critical feedback on methods or results

Carl Michael Baravelli, Rune Blomhoff, Michael Brauer, Benjamin Clarsen, Omid Dadras, Demewoz Haile, Rasmus J Havmoeller, Anne Høyer-Lund, Nityanand Jain, Lars Johansson, Mikk Jürisson, Adnan Kisa, Mika Kivimäki, Ann Kristin Skrindo Knudsen, Ilari Kuitunen, Christian Madsen, Javaid Nauman, Gavin Pereira, Tagli Pitsi, Gerhard Sulo and Eva Warensjö Lemming.

## Drafting the work or revising it critically for important intellectual content

Ayodeji Emmanuel Tope Amobonye, Carl Michael Baravelli, Rune Blomhoff, Anette Kocbach Bølling, Michael Brauer, Benjamin Clarsen, Rasmus J Havmoeller, Anne Høyer-Lund, Nityanand Jain, Mikk Jürisson, Joonas H Kauppila, Adnan Kisa, Mika Kivimäki, Ann Kristin Skrindo Knudsen, Ilari Kuitunen, Christian Madsen, Javaid Nauman, Gavin Pereira, Tagli Pitsi, Pratik Pokharel, Gerhard Sulo, Tommi Juhani Vasankari, and Marcin W Wojewodzic.

## Managing the estimation or publications process

Ann Kristin Skrindo Knudsen
