# Supplementary material for: The burden of dietary risk factors in the Nordic and Baltic countries: a systematic analysis for the Global Burden of Disease Study 2023
Source: Lancet Reg Health Eur. 2025 Nov 25;61:101543. doi: 10.1016/j.lanepe.2025.101543 (PMC12686888; doi:10.1016/j.lanepe.2025.101543)
Supplement: Study Group [file mmc2.docx]

# GBD 2023 Nordic and Baltic Diet Collaborators

| **First Names** | **Last Name** |
| --- | --- |
| Ann Kristin Skrindo | Knudsen^a^ |
| Carl Michael | Baravelli^a^ |
| Christian | Madsen^a^ |
| Benjamin | Clarsen^a,b^ |
| Ayodeji Emmanuel Tope | Amobonye^c^ |
| Michael | Brauer^d, e^ |
| Anette Kocbach | Bølling^f^ |
| Omid | Dadras^g, h^ |
| Demewoz | Haile^e^ |
| Rasmus J | Havmoeller^i^ |
| Anne | Høyer-Lund^j^ |
| Nityanand | Jain^k^ |
| Lars | Johansson^l^ |
| Mikk | Jürisson^m^ |
| Joonas H | Kauppila^n, o^ |
| Adnan | Kisa^p, q^ |
| Mika | Kivimäki^r, s^ |
| Ilari | Kuitunen^t, u^ |
| Javaid | Nauman^w, x^ |
| Gavin | Pereira^y, z^ |
| Tagli | Pitsi^aa^ |
| Pratik | Pokharel^bb, cc^ |
| Tommi Juhani | Vasankari^dd, ee^ |
| Stein Emil | Vollset^a, e^ |
| Eva | Warensjö Lemming^ff, gg^ |
| Marcin W | Wojewodzic^hh, ii^ |
| Rune | Blomhoff^jj^ |

# Affiliations

^a^Department of Disease Burden, Norwegian Institute of Public Health, Bergen, Norway ^b^Department of Sports Medicine, Norwegian School of Sport Sciences, Oslo, Norway ^c^Faculty of Science, Kaunas University of Technology, Kaunas, Lithuania

^d^School of Population and Public Health, University of British Columbia, Vancouver, BC, Canada

^e^Institute for Health Metrics and Evaluation, University of Washington, Seattle, WA, USA ^f^Department of Environmental Health, Norwegian Institute of Public Health, Oslo, Norway

^g^Research Center for Child Psychiatry, University of Turku, Turku, Finland

^h^Iranian Research Center for HIV/AIDS (IRCHA), Tehran University of Medical Sciences, Tehran, Iran

^i^Skaane University Hospital, Skaane County Council, Malmö, Sweden

^j^Department of Preventing NCDs, Norwegian Directorate of Health, Oslo, Norway

^k^Statistics Unit, Riga Stradins University, Riga, Latvia

^l^Department of Public Health, Norwegian Directorate of Health, Oslo, Norway

^m^Institute of Family Medicine and Public Health, University of Tartu, Tartu, Estonia

^n^Surgery Research Unit, University of Oulu, Oulu, Finland

^o^Department of Molecular Medicine and Surgery, Karolinska Institute, Stockholm, Sweden ^p^School of Health Sciences, Kristiania University College, Oslo, Norway

^q^Department of International Health and Sustainable Development, Tulane University, New Orleans, LA, USA

^r^Department of Brain Sciences, University College London, London, UK

^s^Department of Public Health, University of Helsinki, Helsinki, Finland

^t^Department of Pediatrics, Kuopio University Hospital, Kuopio, Finland

^u^Institute of Clinical Medicine, University of Eastern Finland, Kuopio, Finland

^w^College of Medicine and Health Sciences, United Arab Emirates University, Al Ain, United Arab Emirates

^x^Department of Circulation and Medical Imaging, Norwegian University of Science and Technology, Trondheim, Norway

^y^Centre for Fertility and Health, Norwegian Institute of Public Health, Oslo, Norway

^z^School of Population Health, Curtin University, Bentley, WA, Australia

^aa^Department of Nutrition and Physical Activity, National Institute for Health Development, Tallinn, Estonia

^bb^Diet, Cancer, and Health Research Group, Danish Cancer Institute, Copenhagen, Denmark

^cc^Nutrition and Health Innovation Research Institute, Edith Cowan University, Perth, WA, Australia

^dd^UKK Institute, Tampere, Finland;

^ee^Faculty of Medicine and Health Technology, Tampere University, Tampere, Finland ^ff^Department of Food studies, Nutrition and Dietetics, Uppsala University, Uppsala, Sweden

^gg^Department of Surgical Sciences, Uppsala University, Uppsala, Sweden

^hh^Department of Chemical Toxicology, Norwegian Institute of Public Health, Oslo, Norway

^ii^Department of Research, Cancer Registry of Norway, Norwegian Institute of Public Health, Oslo, Norway

^jj^Department of Basic Medical Sciences (Prof. R Blomhoff PhD), University of Oslo, Oslo, Norway
